# Molecular mechanism of Dang-Shen-Yu-Xing decoction against *Mycoplasma bovis* pneumonia based on network pharmacology, molecular docking, molecular dynamics simulations and experimental verification

**DOI:** 10.3389/fvets.2024.1431233

**Published:** 2024-09-24

**Authors:** Mengmeng Yang, Fei Yang, Yanan Guo, Fan Liu, Yong Li, Yanrong Qi, Lei Guo, Shenghu He

**Affiliations:** ^1^College of Animal Science and Technology, Ningxia University, Yinchuan, Ningxia, China; ^2^Institute of Animal Science, Ningxia Academy of Agricultural and Forestry Sciences, Yinchuan, Ningxia, China; ^3^School of Basic Medicine, Ningxia Medical University, Yinchuan, Ningxia, China; ^4^College of Life Science and Technology, Ningxia Polytechnic, Yinchuan, Ningxia, China; ^5^Agricultural and Rural Bureau of Helan County, Yinchuan, Ningxia, China

**Keywords:** Dang-Shen-Yu-Xing decoction, *Mycoplasma bovis* pneumonia, network pharmacology, IL6, IL10

## Abstract

*Mycoplasma bovis* pneumonia is a highly contagious respiratory infection caused by *Mycoplasma bovis*. It is particularly prevalent in calves, posing a significant threat to animal health and leading to substantial economic losses. Dang-Shen-Yu-Xing decoction is often used to treat this condition in veterinary clinics. It exhibits robust anti-inflammatory effects and can alleviate pulmonary fibrosis. However, its mechanism of action remains unclear. Therefore, this study aimed to preliminarily explore the molecular mechanism of Dang-Shen-Yu-Xing decoction for treating *mycoplasma* pneumonia in calves through a combination of network pharmacology, molecular docking, molecular dynamics simulation methods, and experimental validation. The active components and related targets of Dang-Shen-Yu-Xing decoction were extracted from several public databases. Additionally, complex interactions between drugs and targets were explored through network topology, Gene Ontology, and Kyoto Encyclopedia of Genes and Genomes enrichment analyses. Subsequently, the binding affinity of drug to disease-related targets was verified through molecular docking and molecular dynamics simulation. Finally, the pharmacodynamics were verified via animal experiments. The primary network topology analysis revealed two core targets and 10 key active components of Dang-Shen-Yu-Xing decoction against *Mycoplasma bovis* pneumonia. Kyoto Encyclopedia of Genes and Genomes enrichment analysis showed that the mechanism of Dang-Shen-Yu-Xing decoction for treating *mycoplasma bovis* pneumonia involved multiple signaling pathways, with the main pathways including PI3K-Akt and IL17 signaling pathways. Moreover, molecular docking predicted the binding affinity and conformation of the core targets of Dang-Shen-Yu-Xing decoction, IL6, and IL10, with the associated main active ingredients. The results showed a strong binding of the active ingredients to the hub target. Further, molecular docking dynamics simulation revealed three key active components of IL10 induced by Dang-Shen-Yu-Xing decoction against *Mycoplasma bovis* pneumonia. Finally, animal experiments confirmed Dang-Shen-Yu-Xing decoction pharmacodynamics, suggesting that it holds potential as an alternative therapy for treating *mycoplasma bovis* pneumonia.

## 1 Introduction

*Mycoplasma bovis* (*M. bovis*) pneumonia is a highly contagious respiratory disease caused by the bacterium *M. bovis*. This condition predominantly affects calves and young cows, presenting clinical symptoms of fever, coughing, sneezing, and dyspnea. *M. bovis* has caused widespread epidemics owing to the absence of effective vaccine prevention measures and an incomprehensive understanding of its pathogenic mechanism since its discovery in 1961 (1). In the United States, *M.bovis* results in annual losses totaling USD 140 million and can affect up to 70% of cattle in per-feedlot settings (2). In China, more than 10 provinces have reported cases of *M. bovis* infections, and over 40 strains have been identified (3, 4). The prevalence of *M. bovis* pneumonia has increased in relation to beef farm expansion. This respiratory infection typically manifests in feedlots following prolonged transit and is associated with an average mortality rate of approximately 10% (5). However, there are limited treatment options for *M. bovis* pneumonia. Clinical studies have confirmed that macrolide antibiotics are the preferred drugs due to their elevated intracellular concentrations and efficacy against *Mycoplasma*. Nonetheless, macrolides exhibit high toxicity and have side effects, making them susceptible to drug resistance and other adverse conditions (6). Therefore, new treatments for *M. bovis* pneumonia are required, and traditional Chinese medicine (TCM) emerged as a potential alternative.

The treatment of respiratory disorders, particularly *Mycoplasma* pneumonia, using TCM, which has a rich historical background, has been highly successful. This success is primarily attributed to the capacity of herbal medicine to precisely target several molecular mechanisms involved in immunomodulation and anti-inflammatory processes (6). Shuang Huang Lian has remarkable efficacy in treating *Mycoplasma* pneumonia in children, owing primarily to its inhibition of the expression of pro-inflammatory cytokines such as IL6, IL8, and TNF-*α* (7). The Qingfei Tongluo formula mitigates cytokine release, reactive oxygen species (ROS) production, and endoplasmic reticulum stress induced by *Mycoplasma* pneumonia infection by inhibiting the PERK signaling pathway (8). In addition, TCM has been widely utilized during the COVID-19 pandemic in recent years, demonstrating favorable clinical outcomes by exhibiting anti-inflammatory actions, enhancing adaptive immunity, and improving pulmonary fibrosis (9). Therefore, utilizing TCM for pneumonia treatment offers the benefits of multi-component and multi-target actions, along with low susceptibility to drug resistance.

Dang-Shen-Yu-Xing decoction (DSYXD) comprises *Codonopsis*, *Scutellariae*, *Coptidis Rhizoma*, *Poria cocos*, *Isatidis Folium*, *Phellodendri Chinrnsis Cortex*, *Sophorae Flavescentis, Stemonae*, *Houttuyniae*, and *Licorice*. *Codonopsis* is frequently employed to strengthen qi and nourish blood. Furthermore, inulin-type fructans extracted from *Codonopsis* exhibit anti-inflammatory effects by reducing the expression of IL6, TLR4, NF-κB, and TNF-*α* (10). Baicalin—the principal active component of *Scutellariae*—exhibits diverse biological activities and is effective in treating *Mycoplasma* pneumonia infection and inflammation. Furthermore, baicalin also reduced cell apoptosis (11). Other components of DSYXD also demonstrate therapeutic efficacy in respiratory system-related diseases. Among them, *Coptidis Rhizoma* is known for its heat-clearing, detoxifying, antibacterial, and anti-inflammatory properties. Berberine extracted from *Coptidis Rhizoma* inhibits the inflammatory response and fibroblast activation by targeting TNF-*α*, STAT3, IL6, and CCL2, among others (12). In our previous clinical trials, DSYXD demonstrated significant effectiveness in preventing and treating *M. bovis* pneumonia in veterinary clinical settings. Nevertheless, the underlying mechanism through which DSYSD treats *M. bovis* pneumonia remains unclear. Therefore, enhanced comprehension of the regulatory roles of herbs in *Mycoplasma* pneumonia will offer a novel strategy for managing *M. bovis* pneumonia.

Network pharmacology is employed to elucidate the intricate interactions between drugs and disease-related targets by analyzing biological system networks. This approach, which considers multi-components and multi-targets, enables the analysis and prediction of pharmacological mechanisms of drugs (13). It offers the opportunity to systematically explore the relationship between TCM and diseases. Additionally, it has created significant opportunities for the reform and innovation of TCM prescriptions (14). In recent years, molecular dynamics (MD) simulation has emerged as a crucial molecular simulation method in relation to current advancements. It integrates with network pharmacology and molecular docking to enhance the verification and screening of molecular docking results (15, 16). This joint analytical approach will contribute to the comprehensive elucidation of the mechanisms underlying herbal formulas for disease treatment.

The aim of our study was to employ network pharmacology to preliminary explore the active compounds, core targets, and mechanisms by which DSYXD treats *M. bovis* pneumonia. Subsequently, validation was conducted through molecular docking, MD simulations, and animal experiments. The findings of this study provide a theoretical basis for enhanced comprehension of *Mycoplasma* pneumonia pathogenesis in calves and the formulation of novel therapeutic strategies.

## 2 Materials and methods

### 2.1 Materials and reagents

#### 2.1.1 Herbs and reagents

PPLO Broth (lot No. 3018438) was obtained from BD Company, United States. New bovine serum without *Mycoplasma* (lot No. 1128145) was sourced from Gibco. Saline for injection was purchased from Henan Shuanghe Huali Pharmaceutical Co., Ltd., China. *Codonopsis*, *Scutellariae*, *Coptidis Rhizoma*, *Poria cocos*, *Isatidis Folium*, *Phellodendri Chinrnsis Cortex*, *Sophorae Flavescentis*, *Stemonae*, *Houttuyniae*, and *Licorice* were purchased from Ningxia Yinchuan Leming Pharmaceutical Co., Ltd., China. Doxycycline hydrochloride injection was procured from Zhenjiang Witte Pharmaceutical Co., Ltd., China.

#### 2.1.2 *Mycoplasma bovis* culture

*Mycoplasma bovis*, strain NX114 (GenBank accession no. CP135997), was cultured in modified Thiaucourt’s medium for 7 days at 37°C with 5% CO_2_. The medium was supplemented with 20% fetal bovine serum, PPLO broth, 0.04% phenol red, 10% yeast extract, and 800 U/mL penicillin G, with pH adjusted to 7.8. The resuscitated and purified *M. bovis* strain NX114 cultures were extensively subcultured and concentrated to achieve a concentration of 10^10^ CCU/mL for subsequent utilization.

### 2.2 Preparation of DSYXD

*Codonopsis*, *Scutellariae*, *Coptidis Rhizoma*, *Poria cocos*, *Isatidis Folium*, *Phellodendri Chinrnsis Cortex*, *Sophorae Flavescentis*, *Stemonae*, *Houttuyniae*, and *Licorice* were combined at a ratio of 5:4:4:4:4:3:3:3:3:2. Subsequently, they were placed in a Chinese medicine extractor, soaked in water at a ratio of 1:10 (herbs: water) for 60 min, and extracted by simmering for 1 h after boiling. The extract was filtered through gauze. The residual drugs were extracted at a water-to-sample ratio of 8:1 for 45 min. The two decoctions were combined and concentrated to a liquid concentration of 2 g/mL using a rotary evaporator. Subsequently, the samples were stored at 4°C.

### 2.3 Network pharmacology assessment

#### 2.3.1 Screening of active components of DSYXD

Chemical constituents of the 10 herbs were obtained from the Traditional Chinese Medicine Systems Pharmacology database (TCMSP).^1^ These constituents were screened based on oral bioavailability (OB) ≥ 30% and drug-like properties (DL) ≥ 0.18 (17). Compounds that met these criteria were identified as active ingredients.

#### 2.3.2 Identification of targets for DSYXD therapy in *Mycoplasma bovis* pneumonia

In the TCMSP database, target information for the compounds of DSYXD was collected. All collected DSYXD targets were combined and de-duplicated. Subsequently, they were cross-referenced with the UniProt^2^ for correction and transformation into the official gene symbols of *Bos Taurus*. Using the keyword “*Mycoplasma bovis* pneumonia,” the GeneCards,^3^ OMIM,^4^ DrugBank,^5^ CTD,^6^ and PharmGKB^7^ databases were used to collect *M. bovis* pneumonia-related targets, gene duplication, and false positives. DSYXD and *M. bovis* pneumonia-related targets were visualized using the Venn package in R. The cross-targeted genes between the drug and disease were identified as potential targets for DSYXD in treating *M. bovis* pneumonia.

#### 2.3.3 Construction of the regulatory network of DSYXD

The mechanism underlying the treatment of *M. bovis* pneumonia using DSYXD was investigated by building a network between active compounds and cross-targets utilizing Cytoscape 3.7.2 software. This network aids in providing scientific elucidation of the intricate connections among compounds, genes, and diseases.

#### 2.3.4 Construction of protein–protein interaction network and screening of core targets

To investigate the relationship between DSYXD and *M. bovis* pneumonia-related targets, we used the STRING database.^8^ The condition was set to *Bos taurus*, and data with a combined score of ≥0.9 were imported into Cytoscape 3.7.2 software. Subsequently, we constructed a PPI network model. The core target genes of the network were identified by analyzing the betweenness, closeness, degree, eigenvector, local average connectivity-based method (LAC), and network values of the network topology parameters.

#### 2.3.5 Gene Ontology and Kyoto Encyclopedia of Genes and Genomes pathway enrichment analysis

GO and KEGG pathway enrichment analyses were performed using R 4.1.1 software (18), employing the clusterProfiler, org.Bt.eg.db, enrichplot, ggplot2, and pathvie packages. The *p* < 0.05 was considered significant.

#### 2.3.6 Homology modeling

The crystal structure of the IL6 and IL10 proteins from the *Bos taurus* species could not be obtained, necessitating the use of homologous modeling to generate their three-dimensional structures. First, we searched the NCBI database to obtain the IL6 and IL10 protein sequences of bovine origin. Second, the 3D structures of IL6 and IL10 were predicted using the SWISS-Model server^9^ (19). The disordered loops were refined using MODLOOP (20). Subsequently, energy minimization on the modeled structure was conducted using the SWISS-PDB viewer (21). To confirm its stereochemical quality, the reduced model was subjected to various programs from the SAVES server,^10^ including PROCHECK, ERRAT, and ProSA (22–24). The validated model was then visualized and analyzed using PyMOL (25).

#### 2.3.7 Molecular docking

Molecular docking was performed between the core target and its corresponding active component. The 2D structures of the essential compounds were obtained by downloading the SDF file from PubChem^11^ and then refined using ChemBio3D Ultra. Next, the 2D structure file was converted to mol2. The target protein sequence for the receptor was obtained from the NCBI. Additionally, PDB 3D structure files of the target proteins were sourced from the SWISS-MODEL website (see Footnote 9). PyMol software was utilized to remove ligands and water molecules, which were then stored in PDB format. By converting active compounds and core targets to PDBQT format, AutoDockTools 1.5.7 was employed to identify active pockets. Finally, the molecular docking was implemented using AutoDockVina 1.1.2, while the display was handled by PyMol 2.4.0.

#### 2.3.8 Molecular dynamics simulation

MD simulation was performed to evaluate the stability and binding of IL10-luteolin, IL10-quercetin, and IL10-sesamin. The GROMACS 2020 software package was used to conduct MD simulations to enhance credibility and evaluate docking results. AMBER99SB-ILDN force field parameters were used for the protein, and gaff2 generic force field parameters were used for the compound ligand (26, 27). The Sobtop program was used to construct the compound topology and to perform charge fitting using RESP (28). The TIP3P water model was chosen, with atoms in the protein at a minimum distance of 1.0 nm from the edge of the water box, and the system charge was neutralized using sodium or chloride ions based on docking results (29). The two-step equilibrations were carried out for 100 ps at 300 K with a constant number of particles, volume, and temperature (NVT) and a constant number of particles, pressure, and temperature (NPT). The V-rescale temperature coupling method and the Berendsen pressure coupling strategy were employed to maintain constant pressure and temperature in the systems. Upon completion of all simulations, we tallied the number of protein complexes that have been successfully produced across all simulation trajectories. The long-range electrostatic interactions were calculated using the Particle-mesh Ewald method (30). The SHAKE algorithm was employed to rectify all covalent bonds that encompassed hydrogen atoms (31). The Molecular Mechanics Poisson-Boltzmann surface area (MM/PBSA) method was used to compute the binding-free energy (32). The trajectory was obtained from the final 20 ns (80 to 100 ns) of the MD simulation. Trajectory data were saved every 10 ps, and correlation analysis was performed using the trjconv module. Binding free energy calculations for ligands and proteins were performed using the g_MMPBSA method with the Gromacs 2020 program.

### 2.4 Animal experimentation

#### 2.4.1 Animals

Twenty-four 2-month-old female Holstein calves (90–120 kg), were purchased from a cattle farm in Wuzhong City, Ningxia. They underwent a 7-day acclimatization period at the experimental farm. Protocols for general-grade experimental animal operations were followed for all animal-rearing procedures. The Animal Ethics Committee of Ningxia University approved this study (NXU-097). All animal experiments strictly adhered to ethical guidelines to minimize animal numbers and suffering.

#### 2.4.2 Construction of *Mycoplasma bovis* pneumonia model and treatment

Following 7 days of adaptive feeding, six calves were randomly selected as the control group (Con), while the remaining calves were infected with 10^10^ CCU/mL *M. bovis* by nasal drip. The inoculation dose administered was 2 mL/day, and the challenge lasted for 3 days by nasal drip. The control group was administered an equal volume of sterilized normal saline. The groups were isolated and subjected to routine daily examinations for body temperature and clinical symptoms. After 7 days of modeling, serum samples and nasal swabs were obtained from the calves. When these samples tested positive for *M. bovis* infection via PCR and the *Mycoplasma bovis* Ag ELISA kit (Supplementary Table 1; Supplementary Figure 1), the calves were randomly assigned to three groups of six: model group (Mod), DSYXD group, doxycycline group (Dox). The Dox and DSYXD group were administered 0.01 g/kg and 3.5 g/kg, respectively. The control and model groups received an equivalent volume of drinking water. The medication was administered twice daily for 7 days. All animals have free access to water and food. After the last administration, the animals were humanely euthanized under anesthesia to collect biological samples, including serum, lung tissue, and other tissue.

#### 2.4.3 General status assessment

The mental status, coat condition, weight, activity, diet, respiratory condition, and survival rate of the experimental animals were observed and recorded daily.

#### 2.4.4 Spleen index and lung indices

The organ index was calculated by weighing the lung lobes and the spleen. The lung index ratio was determined by dividing lung lobe weight by calf weight, while the spleen index was calculated by dividing spleen weight by calf weight.

#### 2.4.5 Lung histopathological analysis

After 7 days of drug intervention, the lung pathology of the calves was observed. Lung tissues were routinely fixed, paraffin-embedded, sectioned, and then stained with hematoxylin and eosin (H&E) and Masson according to standard protocols. Pathological alterations were detected by H&E staining, while collagen accumulation was visualized using Masson staining. The images were analyzed using Image-Pro Plus 6.0.

### 2.5 Quantitative real-time PCR

Lung total RNA was isolated utilizing the TRIzol Reagent (Life Technologies, United States) and then reverse transcribed into cDNA according to the manufacturer’s instructions for the RNA DNAHiScript II QRT SuperMix (Vazyme, Nanjing, China). The CFX96 real-time PCR equipment (BIO-RAD) and the ChamQ Universal SYBR qPCR Master Mix (Vazyme) were used to perform the qPCR analysis. All gene expression levels were standardized using GAPDH as a reference. Relative gene expressions were calculated using the 2-ΔΔCt technique. The primers were produced by Thermo Fisher Scientific, and are listed in Supplementary Table 2.

### 2.6 Statistical analysis

The data were processed utilizing SPSS 27.0 and subsequently presented as Mean ± SD. One-way ANOVA was used to compare data that followed a normal distribution. The LSD test was used to evaluate the variability, whereas Dunnett’s T3 statistical test was applied in cases of uneven variability. A *p* < 0.05 is regarded as statistically significant.

## 3 Results

### 3.1 Acquisition of potential targets of DSYXD for *Mycoplasma bovis* pneumonia therapy

**Figure 1:**
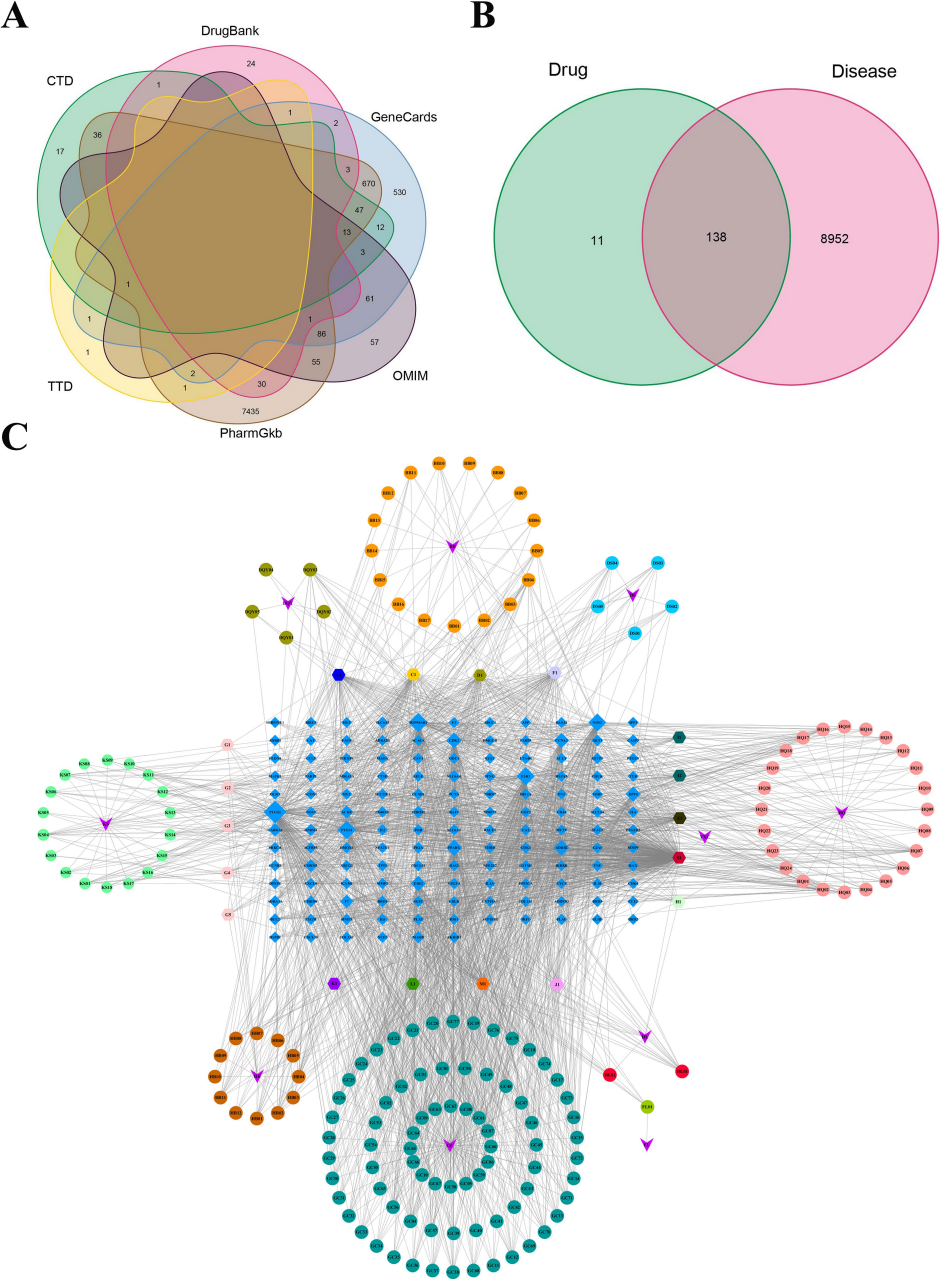
Analysis of potential DSYXD action targets for *Mycoplasma bovis* pneumonia therapy. **(A)** Venn diagram of disease-related targets in six disease databases. **(B)** Venn diagram of potential DSYXD action targets for *Mycoplasma bovis* pneumonia therapy. **(C)** Herb-Compound-Target network of DSYXD. Circles represent components of DSYXS and light blue diamonds represent DSYXS and disease intersection genes. The color and size of the nodes reflect the degree value. Gray lines indicate the interrelationships between compounds and targets.

The chemical components of DSYXD were obtained from TCMSP databases, utilizing screening parameters of OB ≥ 30% and DL ≥ 0.18. Following the removal of duplicate and false-positive components and targets, 275 active components and 149 potential targets for DSYXD action were identified (Supplementary Tables 3, 4). The GeneCards, OMIM, DrugBank, CTD, and TTD databases were utilized to identify and integrate targets associated with *M. bovis* pneumonia. Duplicate targets were eliminated, yielding 9,090 *M. bovis* pneumonia-related targets that are closely linked to its development (Figure 1A). The action targets of DSYXD were cross-referenced with those associated with *M. bovis* pneumonia, resulting in the identification of 138 target genes (Figure 1B; Supplementary Table 5).

### 3.2 “Herb-Compound-Disease-Target” network analysis

To enhance comprehension of the interaction among the 10 herbs, 138 drug-disease potential targets, and 179 related-active compounds, an “Herb-Compound-Disease-Target” network was built, which consisted of 327 nodes and 2,273 edges (Figure 1C). The nodes with more edges in the network exhibited a higher degree value, and their size correspondingly reflected their significance. The top three ingredients identified were quercetin (MOL000098), luteolin (MOL0000066), and kaempferol (MOL000422).

### 3.3 PPI network analysis and screening key targets

**Figure 2:**
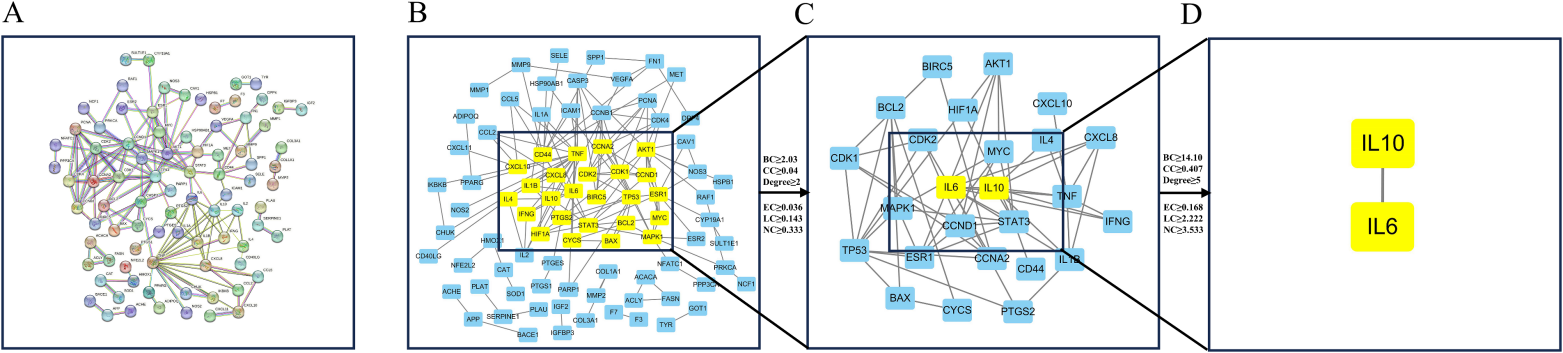
Protein–protein interaction network construction and core target screening. **(A)** PPI network of potential targets for DSYXD therapy of *Mycoplasma bovis* pneumonia in String. **(B)** First screening of core targets (86 nodes, 148 edges); **(C)** Second screening of core targets (25 nodes, 57 edges); **(D)** Third screening of core targets (2 nodes, 1 edge).

The 138 potential targets were inputted into STRING 12.0, resulting in the generation of a PPI network (Figure 2A). Nodes and edges indicate proteins and PPI, respectively. To facilitate further visualization and analysis, we created a unique PPI network comprising 86 nodes and 148 edges in Cytoscape 3.7.2 (Figure 2B). The CytoNCA plug-in was utilized to extract key targets by evaluating the median scores of betweenness, closeness, degree, eigenvector, LAC, and network. Following the initial screening process, we obtained the top 25 key genes and created a new network with Cytoscape (Figure 2C). In the subsequent screening, IL6 and IL10 were identified as significant targets within the anti-*M. bovis* pneumonia pharmacological mechanism of DSYXD (Figure 2D; Supplementary Table 6).

### 3.4 GO and KEGG pathway enrichment analyses

**Figure 3:**
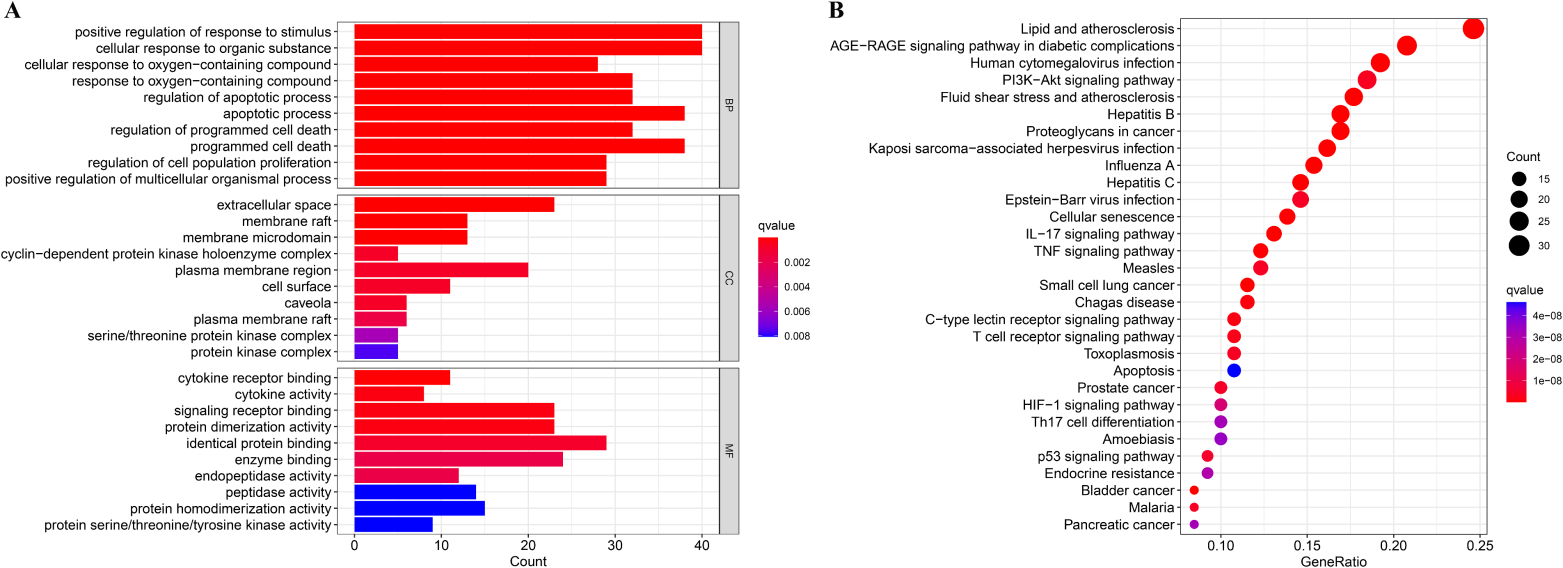
GO and KEGG analysis. **(A)** The top 10 significantly enriched terms (*p* < 0.05) in biological process (BP), cellular component (CC), and molecular function (MF) of GO analysis were selected, **(B)** The top 30 significantly enriched pathways (*p* < 0.05) were selected.

Through GO and KEGG analyses, the biological features and associated signaling pathways of DSYXD - *M. bovis* pneumonia targets were explored. Overall, 965 statistically significant GO terms were identified, including 886, 13, and 66 for biological processes, cellular components, and molecular functions, respectively (Figure 3A; Supplementary Table 7). We identified the most relevant GO terms associated with DSYXD targets as “positive regulation of response to stimulus and cellular response to organic substance,” “extracellular space,” and “identical protein binding.”

KEGG analysis resulted in the enrichment of 166 pathways, with the top 30 shown as bubble plots (Figure 3B; Supplementary Table 8). Based on the findings from KEGG analysis, the potential mechanism of DSYXD for treating *M. bovis* pneumonia may involve pathways, including the AGE-RAGE, PI3K-Akt, IL17, C-type lectin receptor, TNF, and T cell receptor signaling pathways.

### 3.5 Homology modeling

**Figure 4:**
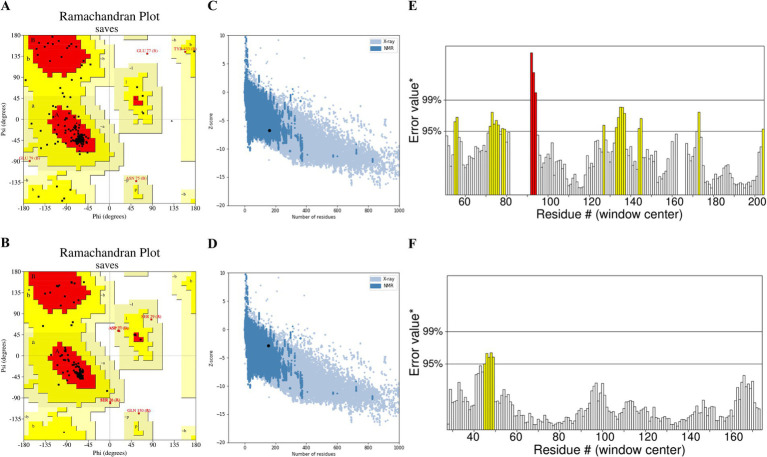
Assessment of the IL6 and IL10 model quality: **(A)** Ramachandran plot illustrating the 3D model of IL6 and **(B)** IL10 generated using PROCHECK. The red, yellow, and white colors indicate the most favored, allowed, and disallowed regions, respectively. **(C)** ERRAT plot showing the quality of the IL6 and **(D)** IL10 models. **(E)** ProSA z-score of the modeled IL6 and **(F)** IL10 structures.

The 3D models of IL6 and IL10 were constructed using the Swiss-Model web server. Subsequently, the 3D structure underwent energy minimization via SWISS-PDB viewer, with disordered segments being refined using ModLoop. The improved model was assessed for quality and accuracy using SAVES server tools. The Ramachandran diagrams of IL6 and IL10 were generated using PROCHECK. The results showed that 75.8 and 92.3% of the amino acid residues resided in the most favorable regions, respectively. Furthermore, 21.6 and 4.9% of the residues were located in additional allowed regions, while 2.0 and 2.8% were situated in generously allowed regions, respectively. Finally, 0.7 and 0.0% of the residues were identified in disallowed regions, respectively (Figures 4A,B; Supplementary Table 9). The modeled structures of IL6 and IL10 were analyzed using the SAVES server. Based on the ERRAT plot, the quality factors of IL6 and IL10 models were 84.06 and 96.59%, respectively (Figures 4C,D). Furthermore, the ProSA server assigned Z-scores of −6.84 and − 2.89 for the IL6 and IL10 modeled structures, respectively. These Z-score values indicate that the predicted IL6 and IL10 structures closely matched the structure of the experimentally determined homologous protein (Figures 4E,F).

### 3.6 Molecular docking

**Figure 5:**
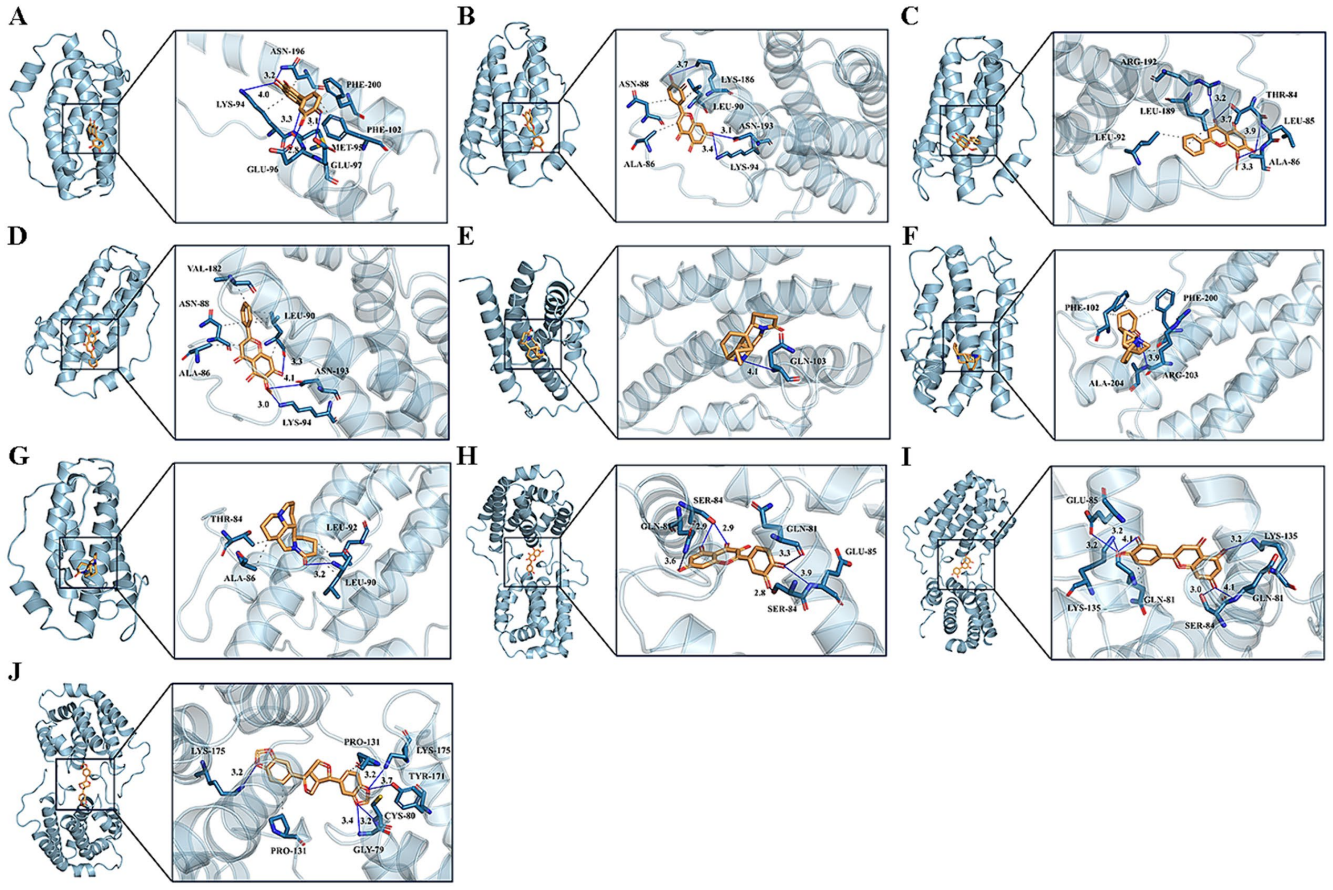
Molecular docking pattern of core compound and core target protein. [The left portion represents the location of core compounds on the protein receptor, and the right represents the name of the specific amino acid that the core compound binds to each protein, and the length of hydrogen bonds **(A)** quercetin-IL6, **(B)** luteolin-IL6, **(C)** wogonin-IL6, **(D)** oroxylin a-IL6, **(E)** matrine-IL6, **(F)** sophocarpine-IL6, **(G)** sophoridine-IL6, **(H)** quercetin-IL10, **(I)** luteolin-IL10, **(J)** sesamin-IL10].

The binding mechanisms of the key drug-disease targets (IL6 and IL10) and their related active components were validated by molecular docking, based on the findings from network pharmacology. Table 1 shows the binding affinities of these components to the target proteins. In molecular docking, the binding-free energy serves as a crucial metric for assessing the stability of drug-target binding; the lower the binding-free energy, the greater the stability of the binding of the drug to the target. The 3D visualization of molecular docking between IL6 and IL10 with their respective active components is shown in Figure 5. The molecular docking revealed successful docking of the compound ligands with the protein receptors. Quercetin, luteolin, and sesamin exhibited the highest binding affinity to the IL10 protein, with values of −7.2, −7.4, and −7.7, respectively (Table 1). In IL10, quercetin formed two hydrogen bonds: one with Gln81 (B) and another with Ser84 (B). Luteolin established four hydrogen bonds: the first with Glu85 (A), the second with Gln81 (A), the third with Ser84 (B), and the final one with Gln81 (B). Sesamin formed four hydrogen bonds: the first with Lys175 (A), the second with Cys80 (B), the third with Gly79 (B), and final one with Lys175 (B; Figure 5).

**Table 1:** The binding energy of compounds and core targets.

Target	compounds	Binding site	Affinity energy
IL6	quercetin	GLU-96, GLU-97	−6.4
	luteolin	LYS-186, ASN-193	−6.5
	wogonin	ARG-192, ASN-88	−6.4
	oroxylin a	LEU-90, ASN-193	−6.8
	matrine	CYS-101	−6.0
	sophocarpine	GLN-103, CYS-101, PHE-200, MET-207	−6.2
	sophoridine	ASN-88	−5.7
IL10	quercetin	GLN-81, SER-84	−7.2
	luteolin	GLN-81, GLU-85, SER-84, GLN-81	−7.4
	sesamin	CYS-80, GLY-79, LYS-175, TYR-77	−7.7

### 3.7 Molecular dynamics simulations

**Figure 6:**
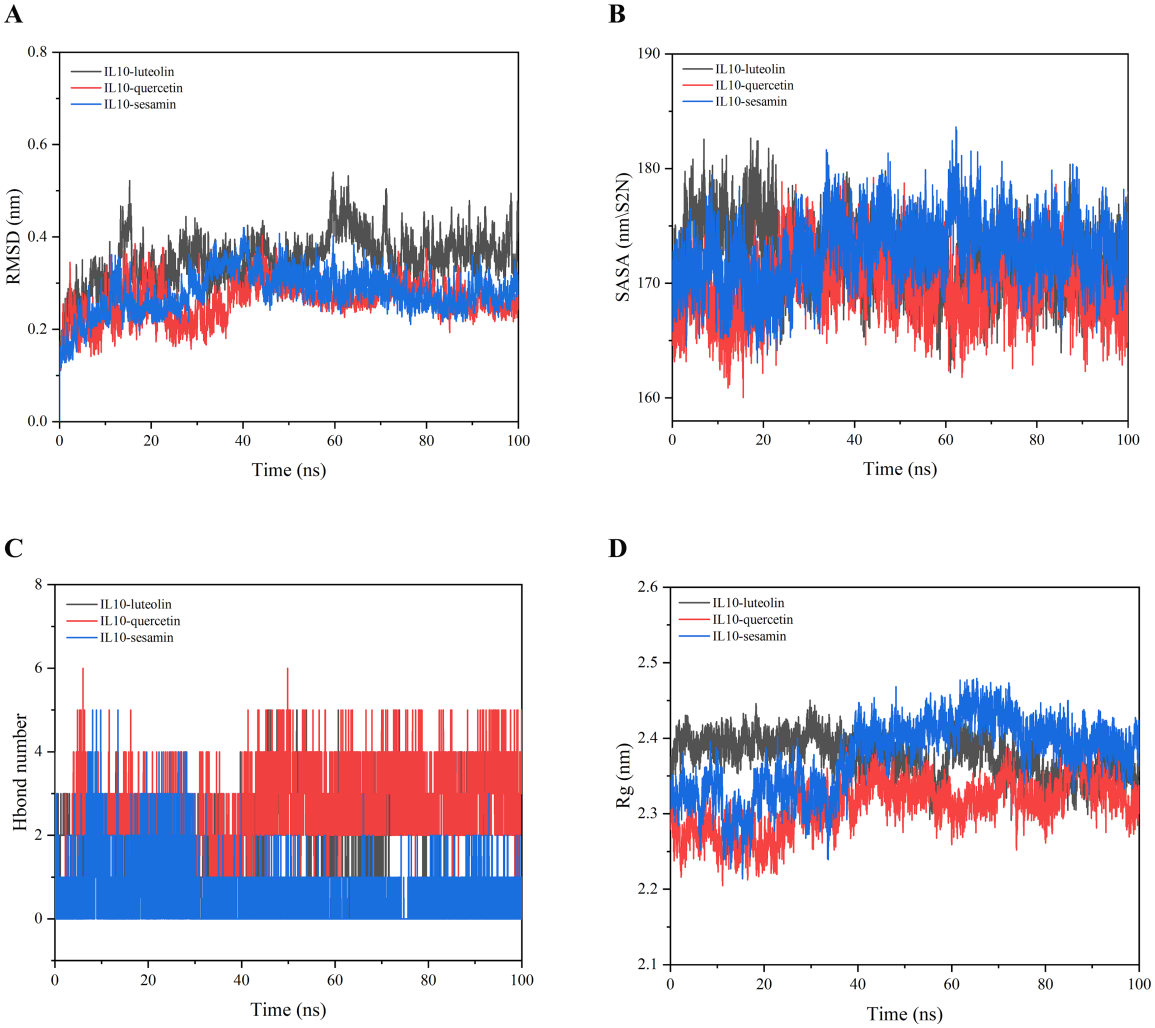
Molecular dynamics simulation of IL10-luteolin, IL10-quercetin, and IL10-sesamin. **(A)** Root mean square deviation (RMSD) values extracted from the protein-ligand docked complexes. **(B)** Solvent accessible surface area (SASA) analysis. **(C)** H-bond formation between IL10 and quercetin, luteolin, and sesamin. **(D)** The compactness of protein structure in terms of the radius of gyration (Rg).

The binding ability of IL10 to quercetin, luteolin, and sesamin was further verified through MD simulation. The RMSD curve illustrates the fluctuation in protein conformation. As shown in Figure 6A, the average RMSD of the IL10-luteolin, IL10-quercetin, and IL10-sesamin complexes was <9 Å, with the RMSD of the IL10-quercetin and IL10-sesamin complexes exhibiting a smaller degree of fluctuation. Furthermore, these two complexes were more stable than IL10-luteolin. In addition, all complexes reached dynamic equilibrium at approximately 20 ns, indicating that the compound effectively matched the target protein and may form a stable complex. No clear fault phenomenon was observed in the RMSD curve. This suggests that the compound did not escape from the protein pocket and may firmly bind to the protein, achieving dynamic equilibrium.

In addition, SASA analysis was conducted to monitor the exposure of the receptor to surrounding solvent molecules during the simulation. The solvent-accessible surface areas of the IL10-quercetin, IL10-luteolin, and IL10-sesamin compounds remained relatively stable throughout the simulation process. This indicates that the binding of small molecules to proteins did not compromise the stability of the proteins, implying that the compounds effectively bound to the proteins (Figure 6B).

The number of hydrogen bonds in the IL10-compound complexes served as an indicator of their binding strength. IL10-quercetin exhibited the highest hydrogen bond density and strength, followed by IL10-luteolin and IL10-sesamin (Figure 6C). The binding free energy served as a metric for assessing the variability and stability of ligand and protein binding modes. MMPBSA calculations showed that IL10-luteolin, IL10-quercetin, and IL10-sesamin exhibited total binding-free energies of −40.219 ± 13.239, −37.607 ± 13.54, and − 124.927 ± 10.26 KJ/mol, respectively. Among these, IL10-sesamin demonstrated the lowest and highest binding free energy and strength, respectively, in line with the findings from molecular docking (Table 2). From the Rg plots, we observed slight fluctuations in the Rg curves of the three complex proteins during the simulation process. Initially, they appeared to increase to a certain extent before experiencing a significant decrease. This may be attributed to the proteins forming more hydrophobic contacts with the compounds after MD simulations, thereby facilitating the formation of more effective interactions within the proteins to match the compounds better. Consequently, this promoted the stability of the complexes (Figure 6D).

**Table 2:** Analysis of protein-ligand molecular mechanics generalized born surface area (MMPBSA; KJ/mol).

Type	IL10-luteolin	IL10-quercetin	IL10-sesamin
Van der Waals energy	−88.541 ± 17.831	−87.33 ± 13.589	−181.838 ± 9.541
Eletrostatic energy	−141.655 ± 38.007	−119.87 ± 18.057	−17.857 ± 5.824
Eletrostatic contribution to solvation	202.504 ± 31.343	180.809 ± 17.895	90.936 ± 9.068
Non-polar contribution to solvation	−12.528 ± 0.554	−11.216 ± 0.763	−16.168 ± 0.629
G_binding energy_	−40.219 ± 13.239	−37.607 ± 13.54	−124.927 ± 10.26

### 3.8 Efficacy of DSYXD in *Mycoplasma bovis* pneumonia model

#### 3.8.1 General conditions of calves

**Figure 7:**
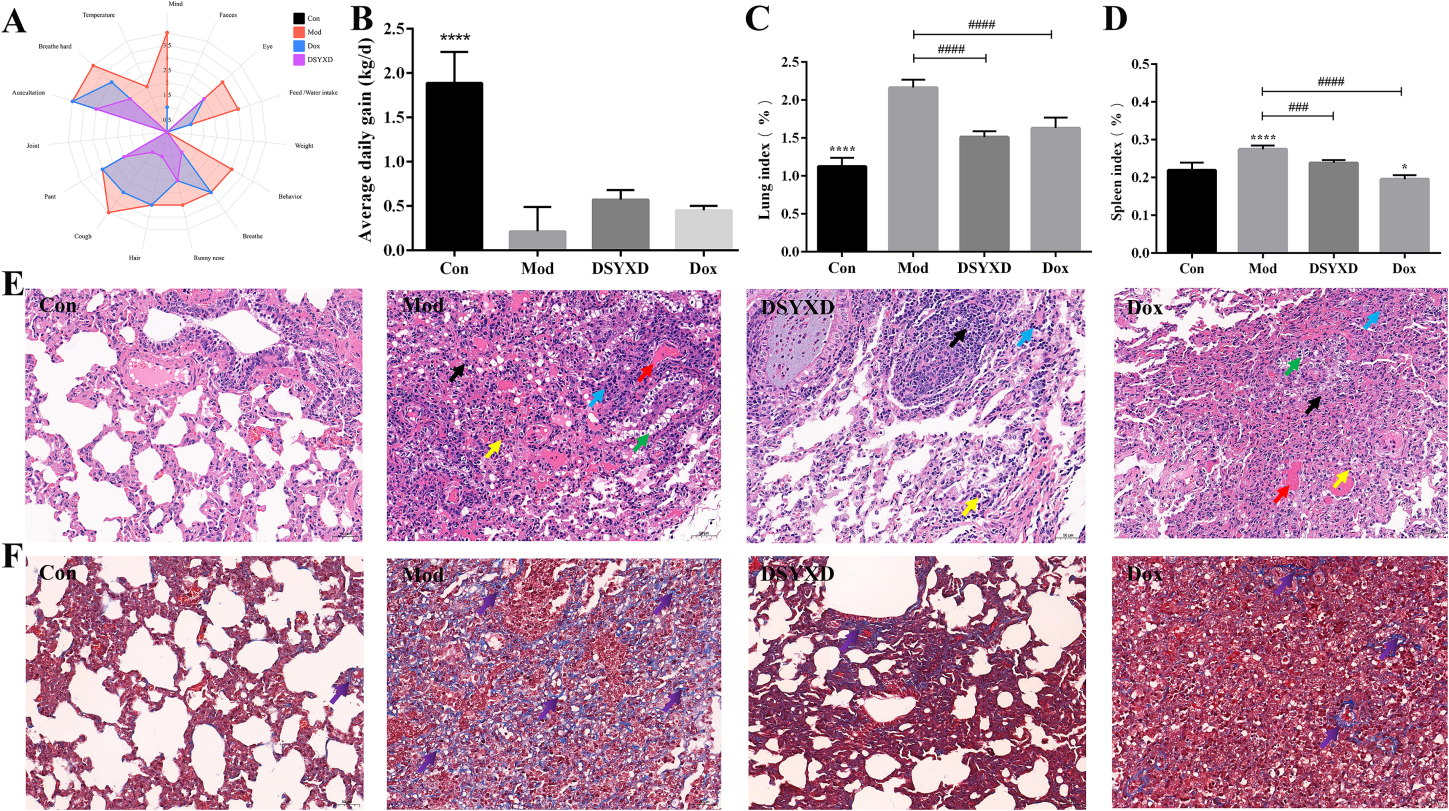
DSYXD effects on calves with *Mycoplasma bovis* pneumonia. **(A)** Radar chart depicting clinical symptoms. **(B)** Daily weight gain comparison among different groups of calves. **(C)** Lung index. **(D)** Spleen index. **(E)** HE staining of lung tissues (×200). **(F)** Masson staining of lung tissues (×200) [Control group (Con); model group (Mod); Dang-Shen-Yu-Xing decoction group (DSYXD); doxycycline group (Dox)].

We established an *M. bovis* pneumonia model to assess the efficacy of DSYXD. Throughout the modeling process of *M. bovis* pneumonia, the calves in the control group exhibited a healthy mental state, normal dietary habits, and no fatalities. During the modeling process, after the third day of the challenge, the pathological model group progressively showed signs of depression, elevated body temperature, increased nasal mucosal secretions, and occasional coughing. By the 7th day, the calves demonstrated clear symptoms, such as panting, abdominal breathing, decreased food intake and dyspnea. After administration, the conditions of calves in the DSYXD-treated group improved (Figure 7A). During the experiment, the body weight of all calves demonstrated an upward trend, with the control group exhibiting the highest daily weight. The model group exhibited the lowest daily weight (*p* < 0.05; Figure 7B; Supplementary Table 10). Furthermore, on days 8 ~ 14 of the experiment, the model group experienced a series of deaths.

#### 3.8.2 DSYXD exerted protective effects against lung injury in *Mycoplasma* pneumonia calves

The study revealed a significant increase in lung and spleen indices of calves with *M. bovis* induced pneumonia. However, notable disparities were identified between the model and treatment groups, indicating the protective effect of the treatment on the lungs and spleens. Moreover, DSYXD exhibited the most effective protection (Figures 7C,D).

In addition, the pathological changes in the lungs were assessed from different perspectives using H&E staining (Figure 7E; Supplementary Figure 2). Calves in the model group showed notable alterations in lung pathomorphology, characterized by extensive necrosis of alveolar epithelial cells and inflammatory cell infiltration, compared with those in the control group. The DSYXD group and Dox group mitigated the above-mentioned pathological damage. In Masson staining, red and blue indicates muscle and collagen fibers, respectively. Compared to that in the control group, a significant accumulation of collagen fibers was observed in the lung tissues of the model group. Conversely, DSYXD significantly ameliorated lung fibrosis (*p* < 0.05; Figure 7F).

#### 3.8.3 Relative expression of IL10 and IL6

**Figure 8:**
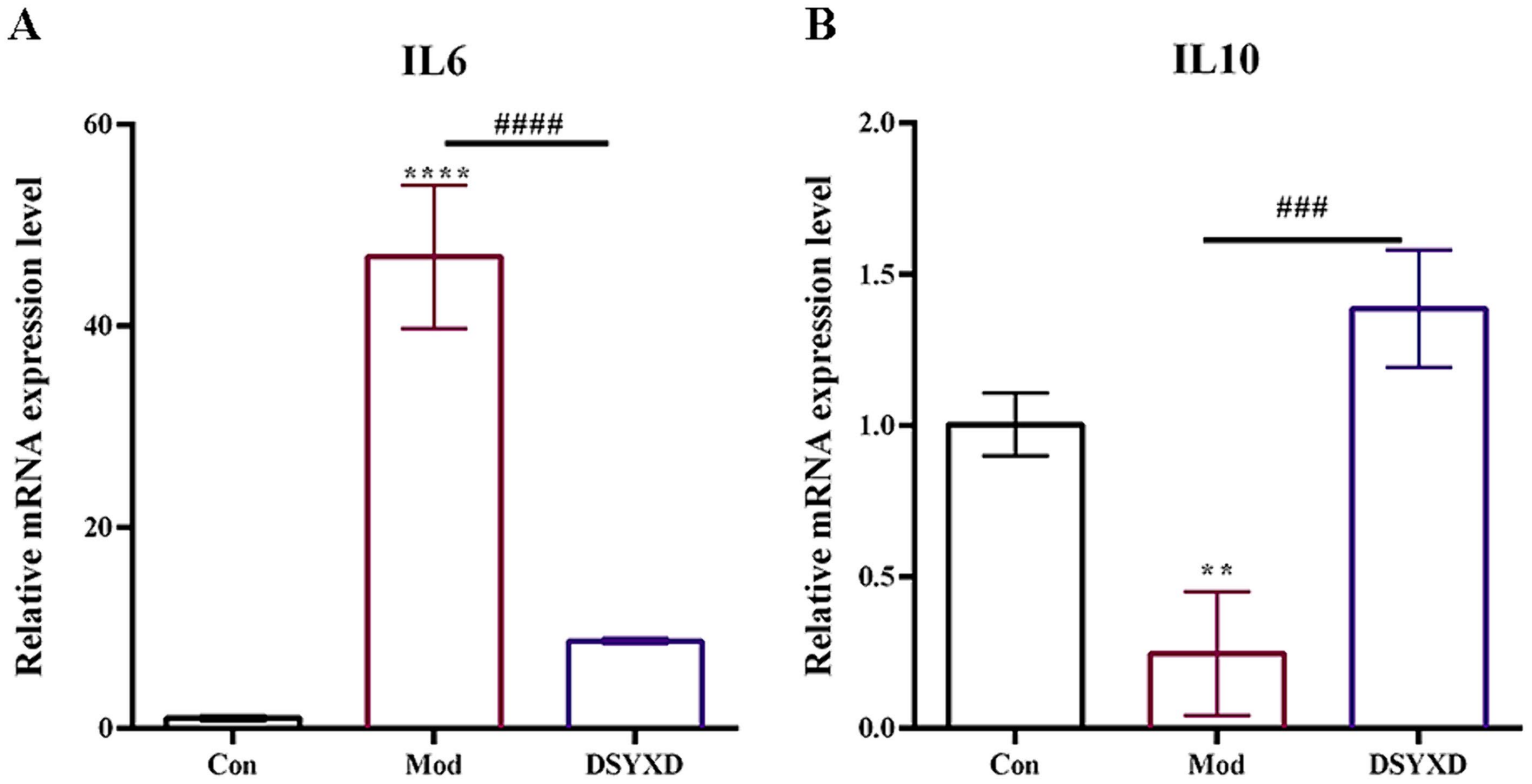
Relative expression of IL6 and IL10 in the lung. **(A)** IL 6. **(B)** IL10. All data are expressed as means ± SD (*n* = 20). *p < 0.05 vs. Control group, #*p* < 0.05 vs. Model group.

Compared to the control group, the result showed that the model group demonstrated increased levels of IL6 and decreased levels of IL10 (Figure 8). The administration of DSYXD resulted in the downregulation and upregulation of the inflammatory cytokines IL6 and IL10, respectively. These findings are consistent with those of the network pharmacology analysis, suggesting that DSYXD reduces lung inflammation in incidents of *M. bovis* pneumonia.

## 4 Discussion

*M. bovis* pneumonia is a common respiratory infectious disease caused by *M. bovis* infection. The etiology of the disease is complex and closely related to factors such as the internal state of the body, environmental conditions, feeding practices, and infectious agents. Nonetheless, the underlying pathogenic mechanism remains incompletely understood (33). Conventional Western medical treatments for *M. bovis* pneumonia have, in some cases, proven ineffective in achieving the desired therapeutic effect and have been associated with an increased incidence of macrolide resistance (34). Nevertheless, the presence of drug remnants in animal-derived products and the inappropriate use of antibiotics, leading to the development of bacteria resistant to drugs, pose a substantial risk to human health (35). Currently, global prohibitions on antibiotics have been implemented to curb their improper use to guarantee the animal-derived food safety (36). Therefore, providing a secure and efficient substitute for antibiotics is imperative to guaranteeing the quality of livestock and poultry products. TCM has attracted extensive scrutiny as an alternative therapy. The use of DSYXD has been extensively in dairy cattle and beef farms in Ningxia, yielding favorable clinical outcomes. However, TCM compounds exhibit varying pharmacological effects involving multiple components, targets, and pathways, rendering it challenging to determine DSYXD composition and the interactions between specific chemical components. Therefore, elucidating the molecular mechanism of its treatment is crucial.

Our findings revealed that most of the active ingredients of DSYXD exert synergistic effects in treating *M. bovis* pneumonia. Additionally, many compounds in DSYXD influenced multiple targets, with some targets overlapping. Among these compounds, quercetin, luteolin, and sesamin emerged as potentially the most active DSYXD compounds in treating *M. bovis* pneumonia, given their effects on various disease-associated targets. Quercetin and luteolin are natural flavonoids, while sesamin belongs to the lignin compound category. The properties of these substances are documented for their ability to fight against oxidation, inflammation, and cancer (37, 38).

Quercetin has demonstrated the ability to affect pro-inflammatory cytokines and protect cells from H_2_O_2_-induced oxidative damage (39). By functioning as an anti-inflammatory compound, quercetin inhibits pathways involved in inflammation, such as HIF1, PI3K/Akt, and JAK–STAT (40–42). Conversely, luteolin exhibits robust antibacterial action and has the ability to eradicate bacterial resistance (43). Additionally, luteolin utilizes the antioxidant characteristics of phenol hydroxyl to eliminate ROS while mitigating inflammatory cell damage. Luteolin exhibits an excellent intervention effect in respiratory disease, significantly inhibiting IL1β and TNF-6 induced by MG, thereby reducing the inflammatory response (44, 45). Furthermore, sesamin can diminish oxidative stress by lowering reactive oxygen species (ROS) and malondialdehyde (MDA) levels and suppressing the secretion of pro-inflammatory cytokines (46).

Previous study findings, including those of ours, provide evidence that quercetin, luteolin, and sesamin may be effective in treating *M. bovis* pneumonia (41). However, other active substances identified through network topology studies should not be neglected. DSYXD exhibits therapeutic effects in *M. bovis* pneumonia owing to synergistic interactions among its components. Hence, further investigation is required to determine whether rationally combined doses of quercetin, luteolin, and sesamin can replicate comparable effects to those of DSYXD. Furthermore, network pharmacological analysis revealed IL10 and IL6 as major targets for DSYXD in treating *M. bovis* pneumonia. The primary components of DSYXD bind to IL6 and IL10, with quercetin, luteolin, and sesamin exhibiting the lowest binding energy and the most stable interaction with IL10. This suggests that DSYXD may boost IL10 expression by binding to it. Unlike Western drugs, DSYXD comprises multiple components that inhibit various pro-inflammatory cytokines (e.g., IL6) while activating anti-inflammatory factors (e.g., IL10). This suggests its potential significance in treating *M. bovis* pneumonia. The most effective strategy against *M. bovis* involves inhibiting its reproduction and colonization (47). *M. bovis* infiltrates the host by selectively binding its membrane protein to cellular receptors, resulting in cellular and tissue damage. Subsequently, it spreads to other organs via the bloodstream (48). IL10—an anti-inflammatory cytokine—is believed to inhibit excessive damage in bacterial and parasitic diseases (49). Furthermore, IL10 suppresses the activation and functionality of several innate and adaptive leukocytes, thereby impeding the production of inflammatory cytokines and preserving host cell integrity (50). IL6—a crucial cytokine—fulfills two distinct roles during inflammation (51). It stimulates the secretion of protective antibodies against extracellular microbial pathogens. Moreover, it is primarily secreted by Th2 cells. IL6 is crucial in the pathogenesis of *MPP* and can serve as an indicator of infection status and disease severity (52). In addition, DSYXD might suppress the development of *M. bovis* pneumonia by modulating the PI3K-Akt and IL17 signaling pathways. PI3K-AKT has been demonstrated to regulate downstream inflammatory cytokines, which are crucial in the inflammatory response (53). However, the anti-inflammatory effects of IL10 are not mediated by PI3K, but its ability to enhance astrocyte survival or induce mast cell proliferation relies on PI3K activation (54). Animal experiment results showed that DSYXD exhibited a significant effect on decreasing lung tissue inflammatory cell infiltration, alveolar dilatation, and bronchial stenosis to varying degrees. The focus of treatment has changed from symptomatic relief to tissue repair. Thus, DSYXD may influence up to 138 drug-disease targets by regulating inflammatory pathways. Furthermore, the results of Masson staining demonstrated that DSYXD effectively alleviated pulmonary fibrosis. Pulmonary fibrosis is a pathological process influenced by various factors, including the inflammatory response, apoptosis, proliferation, and fibroblast activation (55). Additionally, quercetin hinders lung fibrosis progression by acting on multiple mechanisms (56). It upregulates the expression of the FasL receptor and caveolin-1 while inhibiting AKT activation of the apoptotic program in aging fibroblasts (57, 58). In addition, kaempferol can also function as an antifibrotic drug by regulating autophagy and inhibiting inflammatory factor expression (59). These findings indicate that the active components in DSYXD may play a crucial role in the effectiveness of the treatment for *M. bovis* pneumonia. Therefore, further investigation is warranted.

This study still has several limitations. First, the bioavailability of the drug wasn’t verified. Second, we chose representative IL10 and IL6 target genes for pharmacological experimental validation, but we did not test the inhibitory effects of DSYXD on PI3K-Akt, IL17, and other inflammatory pathways associated with *M. bovis* pneumonia. However, our findings also provide a theoretical basis for anti-inflammation for the mechanism of DSYXD and its future experimental verification.

## 5 Conclusion

In this study, we found that DSYXD has a therapeutic effect on *M. bovis* pneumonia by regulating various components, genes, and pathways. By acting on IL6 and IL10 and regulating the PI3K-Akt and IL17 signaling pathways, quercetin, luteolin, and sesamin, found in DSYXD, play a crucial role in its therapeutic effects. *In vivo* experiments further revealed that DSYXD has a significant protective effect on lung tissue injury from *M. bovis* pneumonia. This may be closely related to its ability to enhance anti-inflammatory molecular mechanisms and reduce the formation of pulmonary fibrosis. Therefore, DSYXD can serve as a reliable and efficient pharmaceutical treatment agent to ensure the quality of livestock products.

## Data Availability

The original contributions presented in the study are included in the article/Supplementary materials; further inquiries can be directed to the corresponding authors.

## Footnotes

^1^ https://old.tcmsp-e.com/tcmsp.php

^2^ https://www.uniprot.org/

^3^ https://www.genecards.org/

^4^ https://omim.org/

^5^ https://go.drugbank.com/

^6^ https://ctdbase.org/

^7^ https://www.pharmgkb.org/

^8^ https://cn.string-db.org/

^9^ https://swissmodel.expasy.org/

^10^ http://nihserver.mbi.-ucla.edu/SAVES/

^11^ https://pubchem.ncbi.nlm.nih.gov/
