# Supplementary material for: Molecular mechanism of Dang-Shen-Yu-Xing decoction against Mycoplasma bovis pneumonia based on network pharmacology, molecular docking, molecular dynamics simulations and experimental verification
Source: Front Vet Sci. 2024 Sep 24;11:1431233. doi: 10.3389/fvets.2024.1431233 (PMC11458528; doi:10.3389/fvets.2024.1431233)
Supplement: Supplementary file 1 [file Table_1.docx]

***Supplementary Material***

Molecular mechanism of Dang-Shen-Yu-Xing decoction against *Mycoplasma bovis* pneumonia based on network pharmacology, molecular docking, molecular dynamics simulations and experimental verification

Mengmeng Yang^1,2,3†^, Fei Yang^1,2†^, Yanan Guo^2,*^, Fan Liu^1^, Yong Li^4^, Yanrong Qi^5^, Lei Guo^1^, Shenghu He^1,*^

^1^College of Animal Science and Technology, Ningxia University, Yinchuan, Ningxia 750021, China

^2^Institute of Animal Science, Ningxia Academy of Agricultural and Forestry Sciences, Yinchuan, Ningxia 750002, China

^3^ School of Basic Medicine, Ningxia Medical University, Yinchuan, Ningxia 750001, China

^4^College of Life Science and Technology, Ningxia Polytechnic, Yinchuan, Ningxia 750001, China

^5^Agricultural and Rural Bureau of Helan County, Yinchuan, Ningxia 750200, China

*** Correspondence:**Corresponding Author:
1. Yanan Guo

gyn330@126.com

2. Shenghu He

heshenghu308@163.com

†These authors contributed equally.

# Supplementary Tables and Figures

## Supplementary Tables

**Supplementary Table 1.** The results of *Mycoplasma bovis* Ag ELISA kit

| Animal number | OD_450nm_ value | | | Average | ELISA result |
| --- | --- | --- | --- | --- | --- |
| 1 | 0.681 | 0.752 | 0.608 | 0.680 | + |
| 2 | 0.620 | 0.680 | 0.622 | 0.641 | + |
| 3 | 0.623 | 0.674 | 0.592 | 0.630 | + |
| 4 | 0.640 | 0.561 | 0.582 | 0.594 | + |
| 5 | 0.658 | 0.643 | 0.548 | 0.616 | + |
| 6 | 0.606 | 0.633 | 0.515 | 0.585 | + |
| 7 | 0.653 | 0.703 | 0.685 | 0.680 | + |
| 8 | 0.728 | 0.669 | 0.769 | 0.722 | + |
| 9 | 0.676 | 0.662 | 0.549 | 0.629 | + |
| 10 | 0.645 | 0.606 | 0.665 | 0.639 | + |
| 11 | 0.682 | 0.693 | 0.668 | 0.681 | + |
| 12 | 0.652 | 0.600 | 0.674 | 0.642 | + |
| 13 | 0.616 | 0.635 | 0.666 | 0.639 | + |
| 14 | 0.577 | 0.626 | 0.666 | 0.623 | + |
| 15 | 0.683 | 0.585 | 0.659 | 0.642 | + |
| 16 | 0.666 | 0.679 | 0.600 | 0.648 | + |
| 17 | 0.659 | 0.619 | 0.789 | 0.689 | + |
| 18 | 0.600 | 0.662 | 0.564 | 0.609 | + |
| negative | 0.137 | 0.129 | 0.150 | 0.139 | ¬ |
| positive | 1.681 | 0.976 | 0.833 | 1.163 | + |

Notes: cut off = negative average + 0.15；“-” : OD_450nm_ ＜ Cut off; “+” : OD_450nm_ ＞ Cut off.

**Supplementary Table 2.** Primers for qRT-PCR analysis

| Gene name | Forward primer (5’ to 3’) | Reverse primer (5’ to 3’) |
| --- | --- | --- |
| IL6 | CTTCACAAGCGCCTTCACTC | GTCAGAAGTAGTCTGCCTGG |
| IL10 | TGTTGACCCAGTCTCTGCTG | GGCATCACCTCTTCCAGGTA |

**Supplementary Table 3.** Two hundred and seventy-five active components of DSYXD from TCMSP

| Mol ID | Ingredient name | OB | DL |
| --- | --- | --- | --- |
| MOL000098 | quercetin | 46.43 | 0.28 |
| MOL000006 | luteolin | 36.16 | 0.25 |
| MOL000422 | kaempferol | 41.88 | 0.24 |
| MOL000173 | wogonin | 30.68 | 0.23 |
| MOL003896 | 7-Methoxy-2-methyl isoflavone | 42.56 | 0.2 |
| MOL009374 | 7-methoxy-3-methyl-2,5-dihydroxy-9,10-dihydrophenanthrene | 59 | 0.2 |
| MOL004328 | naringenin | 59.29 | 0.21 |
| MOL000392 | formononetin | 69.67 | 0.21 |
| MOL002714 | baicalein | 33.52 | 0.21 |
| MOL002565 | Medicarpin | 49.22 | 0.34 |
| MOL001558 | sesamin | 56.55 | 0.83 |
| MOL000358 | beta-sitosterol | 36.91 | 0.75 |
| MOL000359 | sitosterol | 36.91 | 0.75 |
| MOL000449 | Stigmasterol | 43.83 | 0.76 |
| MOL005360 | malkangunin | 57.71 | 0.63 |
| MOL005384 | suchilactone | 57.52 | 0.56 |
| MOL009361 | 13,15-Dideoxyaconitine | 34.67 | 0.25 |
| MOL009363 | tuberostemonine C | 55.34 | 0.74 |
| MOL009377 | bisdehydroneotuberostemonine | 51.14 | 0.74 |
| MOL009379 | 2-oxostenine | 72.94 | 0.34 |
| MOL009380 | bisdehydrostemoninine | 38.51 | 0.73 |
| MOL009381 | bisdehydrostemoninine A | 62.64 | 0.68 |
| MOL009382 | bisdehydrostemoninine B | 46.05 | 0.64 |
| MOL009386 | 3,3'-bis-(3,4-dihydro-4-hydroxy-6-methoxy)-2H-1-benzopyran | 52.11 | 0.54 |
| MOL009387 | didehydrotuberostemonine | 51.91 | 0.74 |
| MOL009388 | dihydrostemoninine | 68.01 | 0.72 |
| MOL009394 | stemonamine | 45.19 | 0.35 |
| MOL009409 | oxystemoninine | 42.79 | 0.77 |
| MOL009411 | protostemotinine | 45.99 | 0.75 |
| MOL009414 | sessilifoliamide C | 65.87 | 0.2 |
| MOL009419 | sessilifoliamide H | 43.68 | 0.68 |
| MOL009422 | sessilifoline B | 58.81 | 0.29 |
| MOL009423 | sessilistemonamine A | 40.28 | 0.73 |
| MOL009424 | sessilistemonamine B | 40.64 | 0.73 |
| MOL009430 | stemonamide | 67.46 | 0.38 |
| MOL009431 | stemonine | 81.75 | 0.72 |
| MOL009433 | stemoninine B | 74.77 | 0.73 |
| MOL009434 | stemoninoamide | 66.7 | 0.33 |
| MOL009436 | stemotinine | 38.69 | 0.46 |
| MOL009441 | (3S,3'R,4'R,9S,9aS)-4'-hydroxy-3'-methyl-3-[(2S,4S)-4-methyl-5-oxooxolan-2-yl]spiro[1,2,3,5,6,7,8,9a-octahydropyrrolo[1,2-a]azepine-9,5'-oxolane]-2'-one | 85.52 | 0.38 |
| MOL001006 | poriferasta-7,22E-dien-3beta-ol | 42.98 | 0.76 |
| MOL002140 | Perlolyrine | 65.95 | 0.27 |
| MOL002879 | Diop | 43.59 | 0.39 |
| MOL003036 | ZINC03978781 | 43.83 | 0.76 |
| MOL004355 | Spinasterol | 42.98 | 0.76 |
| MOL004492 | Chrysanthemaxanthin | 38.72 | 0.58 |
| MOL005321 | Frutinone A | 65.9 | 0.34 |
| MOL006554 | Taraxerol | 38.4 | 0.77 |
| MOL006774 | stigmast-7-enol | 37.42 | 0.75 |
| MOL007059 | 3-beta-Hydroxymethyllenetanshiquinone | 32.16 | 0.41 |
| MOL007514 | methyl icosa-11,14-dienoate | 39.67 | 0.23 |
| MOL008391 | 5alpha-Stigmastan-3,6-dione | 33.12 | 0.79 |
| MOL008393 | 7-(beta-Xylosyl)cephalomannine_qt | 38.33 | 0.29 |
| MOL008397 | Daturilin | 50.37 | 0.77 |
| MOL008400 | glycitein | 50.48 | 0.24 |
| MOL008406 | Spinoside A | 39.97 | 0.4 |
| MOL008407 | (8S,9S,10R,13R,14S,17R)-17-[(E,2R,5S)-5-ethyl-6-methylhept-3-en-2-yl]-10,13-dimethyl-1,2,4,7,8,9,11,12,14,15,16,17-dodecahydrocyclopenta[a]phenanthren-3-one | 45.4 | 0.76 |
| MOL008411 | 11-Hydroxyrankinidine | 40 | 0.66 |
| MOL001771 | poriferast-5-en-3beta-ol | 36.91 | 0.75 |
| MOL001781 | Indigo | 38.2 | 0.26 |
| MOL001810 | 6-(3-oxoindolin-2-ylidene)indolo[2,1-b]quinazolin-12-one | 45.28 | 0.89 |
| MOL002308 | Indicaxanthin | 31.79 | 0.22 |
| MOL002309 | indirubin | 48.59 | 0.26 |
| MOL002311 | Glycyrol | 90.78 | 0.67 |
| MOL002318 | C05837 | 66.02 | 0.48 |
| MOL002320 | γ-sitosterol | 36.91 | 0.75 |
| MOL002322 | isovitexin | 31.29 | 0.72 |
| MOL000273 | (2R)-2-[(3S,5R,10S,13R,14R,16R,17R)-3,16-dihydroxy-4,4,10,13,14-pentamethyl-2,3,5,6,12,15,16,17-octahydro-1H-cyclopenta[a]phenanthren-17-yl]-6-methylhept-5-enoic acid | 30.93 | 0.81 |
| MOL000275 | trametenolic acid | 38.71 | 0.8 |
| MOL000276 | 7,9(11)-dehydropachymic acid | 35.11 | 0.81 |
| MOL000279 | Cerevisterol | 37.96 | 0.77 |
| MOL000280 | (2R)-2-[(3S,5R,10S,13R,14R,16R,17R)-3,16-dihydroxy-4,4,10,13,14-pentamethyl-2,3,5,6,12,15,16,17-octahydro-1H-cyclopenta[a]phenanthren-17-yl]-5-isopropyl-hex-5-enoic acid | 31.07 | 0.82 |
| MOL000282 | ergosta-7,22E-dien-3beta-ol | 43.51 | 0.72 |
| MOL000283 | Ergosterol peroxide | 40.36 | 0.81 |
| MOL000285 | (2R)-2-[(5R,10S,13R,14R,16R,17R)-16-hydroxy-3-keto-4,4,10,13,14-pentamethyl-1,2,5,6,12,15,16,17-octahydrocyclopenta[a]phenanthren-17-yl]-5-isopropyl-hex-5-enoic acid | 38.26 | 0.82 |
| MOL000287 | 3beta-Hydroxy-24-methylene-8-lanostene-21-oic acid | 38.7 | 0.81 |
| MOL000289 | pachymic acid | 33.63 | 0.81 |
| MOL000290 | Poricoic acid A | 30.61 | 0.76 |
| MOL000291 | Poricoic acid B | 30.52 | 0.75 |
| MOL000292 | poricoic acid C | 38.15 | 0.75 |
| MOL000296 | hederagenin | 36.91 | 0.75 |
| MOL000300 | dehydroeburicoic acid | 44.17 | 0.83 |
| MOL001484 | Inermine | 75.18 | 0.54 |
| MOL001792 | DFV | 32.76 | 0.18 |
| MOL000211 | Mairin | 55.38 | 0.78 |
| MOL000239 | Jaranol | 50.83 | 0.29 |
| MOL000354 | isorhamnetin | 49.6 | 0.31 |
| MOL003656 | Lupiwighteone | 51.64 | 0.37 |
| MOL000417 | Calycosin | 47.75 | 0.24 |
| MOL004805 | (2S)-2-[4-hydroxy-3-(3-methylbut-2-enyl)phenyl]-8,8-dimethyl-2,3-dihydropyrano[2,3-f]chromen-4-one | 31.79 | 0.72 |
| MOL004806 | euchrenone | 30.29 | 0.57 |
| MOL004808 | glyasperin B | 65.22 | 0.44 |
| MOL004810 | glyasperin F | 75.84 | 0.54 |
| MOL004811 | Glyasperin C | 45.56 | 0.4 |
| MOL004814 | Isotrifoliol | 31.94 | 0.42 |
| MOL004815 | (E)-1-(2,4-dihydroxyphenyl)-3-(2,2-dimethylchromen-6-yl)prop-2-en-1-one | 39.62 | 0.35 |
| MOL004820 | kanzonols W | 50.48 | 0.52 |
| MOL004824 | (2S)-6-(2,4-dihydroxyphenyl)-2-(2-hydroxypropan-2-yl)-4-methoxy-2,3-dihydrofuro[3,2-g]chromen-7-one | 60.25 | 0.63 |
| MOL004827 | Semilicoisoflavone B | 48.78 | 0.55 |
| MOL004828 | Glepidotin A | 44.72 | 0.35 |
| MOL004829 | Glepidotin B | 64.46 | 0.34 |
| MOL004833 | Phaseolinisoflavan | 32.01 | 0.45 |
| MOL004835 | Glypallichalcone | 61.6 | 0.19 |
| MOL004838 | 8-(6-hydroxy-2-benzofuranyl)-2,2-dimethyl-5-chromenol | 58.44 | 0.38 |
| MOL004841 | Licochalcone B | 76.76 | 0.19 |
| MOL004848 | licochalcone G | 49.25 | 0.32 |
| MOL004849 | 3-(2,4-dihydroxyphenyl)-8-(1,1-dimethylprop-2-enyl)-7-hydroxy-5-methoxy-coumarin | 59.62 | 0.43 |
| MOL004855 | Licoricone | 63.58 | 0.47 |
| MOL004856 | Gancaonin A | 51.08 | 0.4 |
| MOL004857 | Gancaonin B | 48.79 | 0.45 |
| MOL004860 | licorice glycoside E | 32.89 | 0.27 |
| MOL004863 | 3-(3,4-dihydroxyphenyl)-5,7-dihydroxy-8-(3-methylbut-2-enyl)chromone | 66.37 | 0.41 |
| MOL004864 | 5,7-dihydroxy-3-(4-methoxyphenyl)-8-(3-methylbut-2-enyl)chromone | 30.49 | 0.41 |
| MOL004866 | 2-(3,4-dihydroxyphenyl)-5,7-dihydroxy-6-(3-methylbut-2-enyl)chromone | 44.15 | 0.41 |
| MOL004879 | Glycyrin | 52.61 | 0.47 |
| MOL004882 | Licocoumarone | 33.21 | 0.36 |
| MOL004883 | Licoisoflavone | 41.61 | 0.42 |
| MOL004884 | Licoisoflavone B | 38.93 | 0.55 |
| MOL004885 | licoisoflavanone | 52.47 | 0.54 |
| MOL004891 | shinpterocarpin | 80.3 | 0.73 |
| MOL004898 | (E)-3-[3,4-dihydroxy-5-(3-methylbut-2-enyl)phenyl]-1-(2,4-dihydroxyphenyl)prop-2-en-1-one | 46.27 | 0.31 |
| MOL004903 | liquiritin | 65.69 | 0.74 |
| MOL004904 | licopyranocoumarin | 80.36 | 0.65 |
| MOL004905 | 3,22-Dihydroxy-11-oxo-delta(12)-oleanene-27-alpha-methoxycarbonyl-29-oic acid | 34.32 | 0.55 |
| MOL004907 | Glyzaglabrin | 61.07 | 0.35 |
| MOL004908 | Glabridin | 53.25 | 0.47 |
| MOL004910 | Glabranin | 52.9 | 0.31 |
| MOL004911 | Glabrene | 46.27 | 0.44 |
| MOL004912 | Glabrone | 52.51 | 0.5 |
| MOL004913 | 1,3-dihydroxy-9-methoxy-6-benzofurano[3,2-c]chromenone | 48.14 | 0.43 |
| MOL004914 | 1,3-dihydroxy-8,9-dimethoxy-6-benzofurano[3,2-c]chromenone | 62.9 | 0.53 |
| MOL004915 | Eurycarpin A | 43.28 | 0.37 |
| MOL004917 | glycyroside | 37.25 | 0.79 |
| MOL004924 | (-)-Medicocarpin | 40.99 | 0.95 |
| MOL004935 | Sigmoidin-B | 34.88 | 0.41 |
| MOL004941 | (2R)-7-hydroxy-2-(4-hydroxyphenyl)chroman-4-one | 71.12 | 0.18 |
| MOL004945 | (2S)-7-hydroxy-2-(4-hydroxyphenyl)-8-(3-methylbut-2-enyl)chroman-4-one | 36.57 | 0.32 |
| MOL004948 | Isoglycyrol | 44.7 | 0.84 |
| MOL004949 | Isolicoflavonol | 45.17 | 0.42 |
| MOL004957 | HMO | 38.37 | 0.21 |
| MOL004959 | 1-Methoxyphaseollidin | 69.98 | 0.64 |
| MOL004961 | Quercetin der. | 46.45 | 0.33 |
| MOL004966 | 3'-Hydroxy-4'-O-Methylglabridin | 43.71 | 0.57 |
| MOL000497 | licochalcone a | 40.79 | 0.29 |
| MOL004974 | 3'-Methoxyglabridin | 46.16 | 0.57 |
| MOL004978 | 2-[(3R)-8,8-dimethyl-3,4-dihydro-2H-pyrano[6,5-f]chromen-3-yl]-5-methoxyphenol | 36.21 | 0.52 |
| MOL004980 | Inflacoumarin A | 39.71 | 0.33 |
| MOL004985 | icos-5-enoic acid | 30.7 | 0.2 |
| MOL004988 | Kanzonol F | 32.47 | 0.89 |
| MOL004989 | 6-prenylated eriodictyol | 39.22 | 0.41 |
| MOL004990 | 7,2',4'-trihydroxy－5-methoxy-3－arylcoumarin | 83.71 | 0.27 |
| MOL004991 | 7-Acetoxy-2-methylisoflavone | 38.92 | 0.26 |
| MOL004993 | 8-prenylated eriodictyol | 53.79 | 0.4 |
| MOL004996 | gadelaidic acid | 30.7 | 0.2 |
| MOL000500 | Vestitol | 74.66 | 0.21 |
| MOL005000 | Gancaonin G | 60.44 | 0.39 |
| MOL005001 | Gancaonin H | 50.1 | 0.78 |
| MOL005003 | Licoagrocarpin | 58.81 | 0.58 |
| MOL005007 | Glyasperins M | 72.67 | 0.59 |
| MOL005008 | Glycyrrhiza flavonol A | 41.28 | 0.6 |
| MOL005012 | Licoagroisoflavone | 57.28 | 0.49 |
| MOL005013 | 18α-hydroxyglycyrrhetic acid | 41.16 | 0.71 |
| MOL005016 | Odoratin | 49.95 | 0.3 |
| MOL005017 | Phaseol | 78.77 | 0.58 |
| MOL005018 | Xambioona | 54.85 | 0.87 |
| MOL005020 | dehydroglyasperins C | 53.82 | 0.37 |
| MOL001454 | berberine | 36.86 | 0.78 |
| MOL001458 | coptisine | 30.67 | 0.86 |
| MOL002636 | Kihadalactone A | 34.21 | 0.82 |
| MOL013352 | Obacunone | 43.29 | 0.77 |
| MOL002641 | Phellavin_qt | 35.86 | 0.44 |
| MOL002643 | delta 7-stigmastenol | 37.42 | 0.75 |
| MOL002644 | Phellopterin | 40.19 | 0.28 |
| MOL002651 | Dehydrotanshinone II A | 43.76 | 0.4 |
| MOL002652 | delta7-Dehydrosophoramine | 54.45 | 0.25 |
| MOL002656 | dihydroniloticin | 36.43 | 0.81 |
| MOL002659 | kihadanin A | 31.6 | 0.7 |
| MOL002660 | niloticin | 41.41 | 0.82 |
| MOL002662 | rutaecarpine | 40.3 | 0.6 |
| MOL002663 | Skimmianin | 40.14 | 0.2 |
| MOL002666 | Chelerythrine | 34.18 | 0.78 |
| MOL002668 | Worenine | 45.83 | 0.87 |
| MOL002670 | Cavidine | 35.64 | 0.81 |
| MOL002671 | Candletoxin A | 31.81 | 0.69 |
| MOL002672 | Hericenone H | 39 | 0.63 |
| MOL002673 | Hispidone | 36.18 | 0.83 |
| MOL000622 | Magnograndiolide | 63.71 | 0.19 |
| MOL000762 | Palmidin A | 35.36 | 0.65 |
| MOL000785 | palmatine | 64.6 | 0.65 |
| MOL000787 | Fumarine | 59.26 | 0.83 |
| MOL000790 | Isocorypalmine | 35.77 | 0.59 |
| MOL001131 | phellamurin_qt | 56.6 | 0.39 |
| MOL001455 | (S)-Canadine | 53.83 | 0.77 |
| MOL002894 | berberrubine | 35.74 | 0.73 |
| MOL005438 | campesterol | 37.58 | 0.71 |
| MOL006401 | melianone | 40.53 | 0.78 |
| MOL006413 | phellochin | 35.41 | 0.82 |
| MOL006422 | thalifendine | 44.41 | 0.73 |
| MOL002897 | epiberberine | 43.09 | 0.78 |
| MOL002903 | (R)-Canadine | 55.37 | 0.77 |
| MOL002904 | Berlambine | 36.68 | 0.82 |
| MOL002907 | Corchoroside A_qt | 104.95 | 0.78 |
| MOL008647 | Moupinamide | 86.71 | 0.26 |
| MOL001689 | acacetin | 34.97 | 0.24 |
| MOL000228 | (2R)-7-hydroxy-5-methoxy-2-phenylchroman-4-one | 55.23 | 0.2 |
| MOL002908 | 5,8,2'-Trihydroxy-7-methoxyflavone | 37.01 | 0.27 |
| MOL002909 | 5,7,2,5-tetrahydroxy-8,6-dimethoxyflavone | 33.82 | 0.45 |
| MOL002910 | Carthamidin | 41.15 | 0.24 |
| MOL002911 | 2,6,2',4'-tetrahydroxy-6'-methoxychaleone | 69.04 | 0.22 |
| MOL002913 | Dihydrobaicalin_qt | 40.04 | 0.21 |
| MOL002914 | Eriodyctiol (flavanone) | 41.35 | 0.24 |
| MOL002915 | Salvigenin | 49.07 | 0.33 |
| MOL002917 | 5,2',6'-Trihydroxy-7,8-dimethoxyflavone | 45.05 | 0.33 |
| MOL002925 | 5,7,2',6'-Tetrahydroxyflavone | 37.01 | 0.24 |
| MOL002926 | dihydrooroxylin A | 38.72 | 0.23 |
| MOL002927 | Skullcapflavone II | 69.51 | 0.44 |
| MOL002928 | oroxylin a | 41.37 | 0.23 |
| MOL002932 | Panicolin | 76.26 | 0.29 |
| MOL002933 | 5,7,4'-Trihydroxy-8-methoxyflavone | 36.56 | 0.27 |
| MOL002934 | NEOBAICALEIN | 104.34 | 0.44 |
| MOL002937 | DIHYDROOROXYLIN | 66.06 | 0.23 |
| MOL000525 | Norwogonin | 39.4 | 0.21 |
| MOL000552 | 5,2'-Dihydroxy-6,7,8-trimethoxyflavone | 31.71 | 0.35 |
| MOL000073 | ent-Epicatechin | 48.96 | 0.24 |
| MOL001490 | bis[(2S)-2-ethylhexyl] benzene-1,2-dicarboxylate | 43.59 | 0.35 |
| MOL001506 | Supraene | 33.55 | 0.42 |
| MOL008206 | Moslosooflavone | 44.09 | 0.25 |
| MOL010415 | 11,13-Eicosadienoic acid, methyl ester | 39.28 | 0.23 |
| MOL012245 | 5,7,4'-trihydroxy-6-methoxyflavanone | 36.63 | 0.27 |
| MOL012246 | 5,7,4'-trihydroxy-8-methoxyflavanone | 74.24 | 0.26 |
| MOL012266 | rivularin | 37.94 | 0.37 |
| MOL001040 | (2R)-5,7-dihydroxy-2-(4-hydroxyphenyl)chroman-4-one | 42.36 | 0.21 |
| MOL003542 | 8-Isopentenyl-kaempferol | 38.04 | 0.39 |
| MOL003627 | sophocarpine | 64.26 | 0.25 |
| MOL003648 | Inermin | 65.83 | 0.54 |
| MOL003673 | Wighteone | 42.8 | 0.36 |
| MOL003676 | Sophoramine | 42.16 | 0.25 |
| MOL003680 | sophoridine | 60.07 | 0.25 |
| MOL004580 | cis-Dihydroquercetin | 66.44 | 0.27 |
| MOL005100 | 5,7-dihydroxy-2-(3-hydroxy-4-methoxyphenyl)chroman-4-one | 47.74 | 0.27 |
| MOL005944 | matrine | 63.77 | 0.25 |
| MOL006561 | (+)-14alpha-hydroxymatrine | 35.73 | 0.29 |
| MOL006562 | (+)-7,11-dehydromatrine,(leontalbinine) | 62.08 | 0.25 |
| MOL006563 | (+)-9alpha-hydroxymatrine | 32.04 | 0.29 |
| MOL006564 | (+)-allomatrine | 58.87 | 0.25 |
| MOL006565 | AIDS211310 | 68.68 | 0.25 |
| MOL006566 | (+)-lehmannine | 58.34 | 0.25 |
| MOL006568 | isosophocarpine | 61.57 | 0.25 |
| MOL006569 | (-)-14beta-hydroxymatrine | 37.26 | 0.29 |
| MOL006570 | (-)-9alpha-hydroxysophoramine | 35.23 | 0.29 |
| MOL006571 | anagyrine | 62.01 | 0.24 |
| MOL006572 | 1,4-diazaindan-type,alkaloid,flavascensine | 34.64 | 0.24 |
| MOL006573 | 13,14-dehydrosophoridine | 65.34 | 0.25 |
| MOL006582 | 5α,9α-dihydroxymatrine | 40.93 | 0.32 |
| MOL006583 | 7,11-dehydromatrine | 44.43 | 0.25 |
| MOL006596 | Glyceollin | 97.27 | 0.76 |
| MOL003347 | hyperforin | 44.03 | 0.6 |
| MOL006604 | (2S)-7-hydroxy-2-(4-hydroxyphenyl)-5-methoxy-8-(3-methylbut-2-enyl)chroman-4-one | 48.09 | 0.39 |
| MOL006613 | kushenin | 47.62 | 0.38 |
| MOL006619 | kushenol J | 51.39 | 0.74 |
| MOL006620 | kushenol J_qt | 50.86 | 0.24 |
| MOL006622 | kushenol O | 42.41 | 0.76 |
| MOL006623 | kushenol,t | 51.28 | 0.64 |
| MOL006626 | leachianone,g | 60.97 | 0.4 |
| MOL006627 | Lehmanine | 62.23 | 0.25 |
| MOL006628 | (+)-Lupanine | 52.71 | 0.24 |
| MOL006630 | Norartocarpetin | 54.93 | 0.24 |
| MOL000456 | Phaseolin | 78.2 | 0.73 |
| MOL006649 | sophranol | 55.42 | 0.28 |
| MOL006650 | (-)-Maackiain-3-O-glucosyl-6'-O-malonate | 48.69 | 0.52 |
| MOL006652 | trifolrhizin | 48.53 | 0.74 |
| MOL003851 | Isoramanone | 39.97 | 0.51 |
| MOL004345 | 1-methyl-2-nonacosyl-4-quinolone | 31.54 | 0.5 |
| MOL004350 | Ruvoside_qt | 36.12 | 0.76 |
| MOL004351 | C09747 | 37.28 | 0.25 |

**Supplementary Table 4.** One hundred and forty-nine targets of DSYXD

| NO. | Target |
| --- | --- |
| 1 | PTGS2 |
| 2 | CCND1 |
| 3 | IL10 |
| 4 | FASN |
| 5 | ACACA |
| 6 | NOS3 |
| 7 | ECE1 |
| 8 | ACADM |
| 9 | ACOX1 |
| 10 | ACLY |
| 11 | HADHB |
| 12 | PTGS1 |
| 13 | HSP90AB1 |
| 14 | DRD1 |
| 15 | CHRM3 |
| 16 | CHRM4 |
| 17 | ADRA1A |
| 18 | CHRM2 |
| 19 | ADRB2 |
| 20 | SLC6A4 |
| 21 | OPRM1 |
| 22 | GABRA1 |
| 23 | BCL2 |
| 24 | BAX |
| 25 | CASP3 |
| 26 | PRKCA |
| 27 | NOS2 |
| 28 | ESR1 |
| 29 | PPARG |
| 30 | SLC6A3 |
| 31 | ESR2 |
| 32 | DPP4 |
| 33 | CDK2 |
| 34 | MAOB |
| 35 | CCNA2 |
| 36 | CALM |
| 37 | PKIA |
| 38 | ACHE |
| 39 | DPEP1 |
| 40 | IL4 |
| 41 | ATP5F1B |
| 42 | ADRA2A |
| 43 | SLC6A2 |
| 44 | AKR1B1 |
| 45 | PLAU |
| 46 | LTA4H |
| 47 | MAOA |
| 48 | ADRB1 |
| 49 | F7 |
| 50 | DRD2 |
| 51 | AKT1 |
| 52 | VEGFA |
| 53 | MMP2 |
| 54 | MMP9 |
| 55 | MAPK1 |
| 56 | CDK4 |
| 57 | TNF |
| 58 | IL6 |
| 59 | TP53 |
| 60 | APP |
| 61 | MMP1 |
| 62 | PCNA |
| 63 | HMOX1 |
| 64 | ICAM1 |
| 65 | BIRC5 |
| 66 | IL2 |
| 67 | CCNB1 |
| 68 | TYR |
| 69 | IFNG |
| 70 | GSTP1 |
| 71 | SLC2A4 |
| 72 | CD40LG |
| 73 | PTGES |
| 74 | MET |
| 75 | CA2 |
| 76 | MMP13 |
| 77 | CCL5 |
| 78 | IKBKB |
| 79 | LYZ1 |
| 80 | PYGM |
| 81 | NCF1 |
| 82 | OLR1 |
| 83 | CDK1 |
| 84 | SELE |
| 85 | HAS2 |
| 86 | PSMD3 |
| 87 | PPP3CA |
| 88 | GSTM1 |
| 89 | LDLR |
| 90 | SOD1 |
| 91 | CAT |
| 92 | MTTP |
| 93 | HMGCR |
| 94 | CYP19A1 |
| 95 | ABCC1 |
| 96 | ADIPOQ |
| 97 | GOT1 |
| 98 | ABAT |
| 99 | BACE1 |
| 100 | STAT3 |
| 101 | EIF6 |
| 102 | POR |
| 103 | ODC1 |
| 104 | RAF1 |
| 105 | HIF1A |
| 106 | HSPA5 |
| 107 | CAV1 |
| 108 | MYC |
| 109 | F3 |
| 110 | GJA1 |
| 111 | IL1B |
| 112 | CCL2 |
| 113 | PTGER3 |
| 114 | CXCL8 |
| 115 | PRKCB |
| 116 | HSPB1 |
| 117 | SULT1E1 |
| 118 | PLAT |
| 119 | THBD |
| 120 | SERPINE1 |
| 121 | COL1A1 |
| 122 | IL1A |
| 123 | ABCG2 |
| 124 | NFE2L2 |
| 125 | PARP1 |
| 126 | COL3A1 |
| 127 | CXCL11 |
| 128 | CLDN4 |
| 129 | HSF1 |
| 130 | CXCL10 |
| 131 | CHUK |
| 132 | SPP1 |
| 133 | ACP3 |
| 134 | CTSD |
| 135 | IGFBP3 |
| 136 | IGF2 |
| 137 | IRF1 |
| 138 | RASA1 |
| 139 | DRD5 |
| 140 | ADRA2B |
| 141 | FN1 |
| 142 | CYCS |
| 143 | NFATC1 |
| 144 | TDRD7 |
| 145 | FABP5 |
| 146 | APOD |
| 147 | HPSE |
| 148 | IER3IP1 |
| 149 | CD44 |

**Supplementary Table 5.** One hundred and thirty-eight targets of DSYXD-*Mycoplasma bovis* pneumonia

| NO | Target |
| --- | --- |
| 1 | CCND1 |
| 2 | IL10 |
| 3 | FASN |
| 4 | ACACA |
| 5 | NOS3 |
| 6 | ECE1 |
| 7 | ACOX1 |
| 8 | ACLY |
| 9 | PTGS1 |
| 10 | PTGS2 |
| 11 | HSP90AB1 |
| 12 | DRD1 |
| 13 | CHRM3 |
| 14 | CHRM4 |
| 15 | ADRA1A |
| 16 | CHRM2 |
| 17 | ADRB2 |
| 18 | SLC6A4 |
| 19 | OPRM1 |
| 20 | GABRA1 |
| 21 | BCL2 |
| 22 | BAX |
| 23 | CASP3 |
| 24 | PRKCA |
| 25 | NOS2 |
| 26 | ESR1 |
| 27 | PPARG |
| 28 | SLC6A3 |
| 29 | ESR2 |
| 30 | DPP4 |
| 31 | CDK2 |
| 32 | MAOB |
| 33 | CCNA2 |
| 34 | PKIA |
| 35 | ACHE |
| 36 | DPEP1 |
| 37 | IL4 |
| 38 | ATP5F1B |
| 39 | ADRA2A |
| 40 | SLC6A2 |
| 41 | AKR1B1 |
| 42 | PLAU |
| 43 | LTA4H |
| 44 | MAOA |
| 45 | ADRB1 |
| 46 | F7 |
| 47 | DRD2 |
| 48 | AKT1 |
| 49 | VEGFA |
| 50 | MMP2 |
| 51 | MMP9 |
| 52 | MAPK1 |
| 53 | CDK4 |
| 54 | TNF |
| 55 | IL6 |
| 56 | TP53 |
| 57 | APP |
| 58 | MMP1 |
| 59 | PCNA |
| 60 | HMOX1 |
| 61 | ICAM1 |
| 62 | BIRC5 |
| 63 | IL2 |
| 64 | CCNB1 |
| 65 | TYR |
| 66 | IFNG |
| 67 | GSTP1 |
| 68 | SLC2A4 |
| 69 | CD40LG |
| 70 | PTGES |
| 71 | MET |
| 72 | CA2 |
| 73 | MMP13 |
| 74 | CCL5 |
| 75 | IKBKB |
| 76 | PYGM |
| 77 | NCF1 |
| 78 | OLR1 |
| 79 | CDK1 |
| 80 | SELE |
| 81 | HAS2 |
| 82 | PPP3CA |
| 83 | GSTM1 |
| 84 | LDLR |
| 85 | SOD1 |
| 86 | CAT |
| 87 | MTTP |
| 88 | HMGCR |
| 89 | CYP19A1 |
| 90 | ABCC1 |
| 91 | ADIPOQ |
| 92 | GOT1 |
| 93 | BACE1 |
| 94 | STAT3 |
| 95 | POR |
| 96 | ODC1 |
| 97 | RAF1 |
| 98 | HIF1A |
| 99 | HSPA5 |
| 100 | CAV1 |
| 101 | MYC |
| 102 | F3 |
| 103 | GJA1 |
| 104 | IL1B |
| 105 | CCL2 |
| 106 | PTGER3 |
| 107 | CXCL8 |
| 108 | HSPB1 |
| 109 | SULT1E1 |
| 110 | PLAT |
| 111 | THBD |
| 112 | SERPINE1 |
| 113 | COL1A1 |
| 114 | IL1A |
| 115 | ABCG2 |
| 116 | NFE2L2 |
| 117 | PARP1 |
| 118 | COL3A1 |
| 119 | CXCL11 |
| 120 | CLDN4 |
| 121 | HSF1 |
| 122 | CXCL10 |
| 123 | CHUK |
| 124 | SPP1 |
| 125 | CTSD |
| 126 | IGFBP3 |
| 127 | IGF2 |
| 128 | IRF1 |
| 129 | RASA1 |
| 130 | DRD5 |
| 131 | ADRA2B |
| 132 | FN1 |
| 133 | CYCS |
| 134 | NFATC1 |
| 135 | TDRD7 |
| 136 | FABP5 |
| 137 | IER3IP1 |
| 138 | CD44 |

**Supplementary Table 6.** Topological parameter information for PPI

| NO | name | Betweenness | Closeness | Degree | Eigenvector | LAC | Network |
| --- | --- | --- | --- | --- | --- | --- | --- |
| 1 | TNF | 1296.308153 | 0.041483651 | 17 | 0.194899485 | 2.235294118 | 11.7047619 |
| 2 | AKT1 | 544.1088745 | 0.041483651 | 11 | 0.23610267 | 1.818181818 | 4.430555556 |
| 3 | TP53 | 575.7476912 | 0.041282176 | 11 | 0.251334786 | 1.272727273 | 3.73015873 |
| 4 | ESR1 | 420.4325397 | 0.04136253 | 10 | 0.260091007 | 2.6 | 6.041666667 |
| 5 | CCND1 | 317.7865079 | 0.041142304 | 9 | 0.316856474 | 3.333333333 | 5.163095238 |
| 6 | STAT3 | 969.4188312 | 0.041789577 | 9 | 0.255975872 | 2.222222222 | 3.160714286 |
| 7 | MAPK1 | 638.6628427 | 0.041524182 | 9 | 0.195646286 | 1.333333333 | 2.833333333 |
| 8 | CCNA2 | 70.35079365 | 0.040786948 | 8 | 0.267974198 | 4.25 | 6.523809524 |
| 9 | IL10 | 295.6637807 | 0.041382668 | 8 | 0.16108878 | 3.25 | 5.580952381 |
| 10 | IL6 | 312.8196248 | 0.041443198 | 8 | 0.162349537 | 3 | 5.171428571 |
| 11 | CDK1 | 52.28888889 | 0.040767386 | 7 | 0.243592024 | 3.714285714 | 4.933333333 |
| 12 | CDK2 | 42.93015873 | 0.040553435 | 7 | 0.251723528 | 4.285714286 | 5.633333333 |
| 13 | CD44 | 673.2666667 | 0.041082649 | 7 | 0.057748448 | 0.285714286 | 1.333333333 |
| 14 | CASP3 | 790.8845599 | 0.041544477 | 6 | 0.127337605 | 0 | 0 |
| 15 | CCNB1 | 13.54285714 | 0.04005655 | 6 | 0.193830281 | 3.666666667 | 4.816666667 |
| 16 | CXCL8 | 30.41645022 | 0.040669856 | 6 | 0.111104384 | 2.666666667 | 3.8 |
| 17 | PCNA | 7.176984127 | 0.040227165 | 6 | 0.21892184 | 4.333333333 | 5.4 |
| 18 | IL1B | 74.21139971 | 0.040845747 | 6 | 0.117262252 | 3 | 4.333333333 |
| 19 | MYC | 11.7452381 | 0.0411224 | 5 | 0.190087095 | 3.2 | 4 |
| 20 | HIF1A | 246.674531 | 0.041162228 | 5 | 0.123578534 | 0.8 | 1.75 |
| 21 | CDK4 | 6.776984127 | 0.040208136 | 5 | 0.187184691 | 3.6 | 4.5 |
| 22 | BCL2 | 31.69047619 | 0.040669856 | 4 | 0.081528723 | 1.5 | 2.5 |
| 23 | BIRC5 | 127.6793651 | 0.040669856 | 4 | 0.124774121 | 1.5 | 2 |
| 24 | CXCL10 | 124.6666667 | 0.040341718 | 4 | 0.046042498 | 1 | 1.666666667 |
| 25 | IL4 | 124 | 0.040572792 | 4 | 0.066347376 | 1.5 | 2 |
| 26 | FN1 | 136.5880952 | 0.040075436 | 4 | 0.01403172 | 0.5 | 1.333333333 |
| 27 | IFNG | 2.066666667 | 0.040630975 | 4 | 0.089211419 | 2.5 | 3.333333333 |
| 28 | IL2 | 1.333333333 | 0.040572792 | 4 | 0.076684684 | 2.5 | 3.333333333 |
| 29 | PTGS2 | 328.3275613 | 0.041003377 | 4 | 0.081533358 | 0.5 | 0.666666667 |
| 30 | CAV1 | 0 | 0.040534096 | 3 | 0.087864161 | 2 | 3 |
| 31 | NOS3 | 0 | 0.040534096 | 3 | 0.087864161 | 2 | 3 |
| 32 | BAX | 12.03809524 | 0.04041845 | 3 | 0.055956554 | 1.333333333 | 2 |
| 33 | CYCS | 51.80714286 | 0.040514776 | 3 | 0.039791636 | 0.666666667 | 1 |
| 34 | CCL2 | 1.333333333 | 0.040322581 | 3 | 0.052753091 | 1.333333333 | 2 |
| 35 | CCL5 | 1.333333333 | 0.040322581 | 3 | 0.052753091 | 1.333333333 | 2 |
| 36 | MMP9 | 131.4380952 | 0.04005655 | 3 | 0.012365118 | 0 | 0 |
| 37 | VEGFA | 97.41666667 | 0.040208136 | 3 | 0.022561818 | 0 | 0 |
| 38 | IL1A | 0 | 0.040611562 | 3 | 0.07113608 | 2 | 3 |
| 39 | PRKCA | 135.9666667 | 0.040437678 | 3 | 0.036425024 | 0 | 0 |
| 40 | ACACA | 0 | 0.011904762 | 2 | 0 | 1 | 2 |
| 41 | FASN | 0 | 0.011904762 | 2 | 0 | 1 | 2 |
| 42 | ACLY | 0 | 0.011904762 | 2 | 0 | 1 | 2 |
| 43 | APP | 2 | 0.011904762 | 2 | 0 | 0 | 0 |
| 44 | PPARG | 124 | 0.040303461 | 2 | 0.029873218 | 0 | 0 |
| 45 | RAF1 | 20.9047619 | 0.04041845 | 2 | 0.041000448 | 0 | 0 |
| 46 | MET | 36.01031746 | 0.040572792 | 2 | 0.044201843 | 0 | 0 |
| 47 | PARP1 | 13.63189033 | 0.040845747 | 2 | 0.056858521 | 0 | 0 |
| 48 | CAT | 4 | 0.012046485 | 2 | 0 | 0 | 0 |
| 49 | HMOX1 | 4 | 0.012046485 | 2 | 0 | 0 | 0 |
| 50 | ICAM1 | 210.5531746 | 0.040826129 | 2 | 0.037891179 | 0 | 0 |
| 51 | SPP1 | 0 | 0.039943609 | 2 | 0.010803161 | 1 | 2 |
| 52 | CHUK | 0 | 0.04028436 | 2 | 0.034350943 | 1 | 2 |
| 53 | IKBKB | 0 | 0.04028436 | 2 | 0.034350943 | 1 | 2 |
| 54 | COL1A1 | 2 | 0.011904762 | 2 | 0 | 0 | 0 |
| 55 | CYP19A1 | 124 | 0.040189125 | 2 | 0.040026423 | 0 | 0 |
| 56 | ESR2 | 0 | 0.040514776 | 2 | 0.068541139 | 1 | 2 |
| 57 | HSP90AB1 | 0 | 0.040437678 | 2 | 0.056306358 | 1 | 2 |
| 58 | NFATC1 | 124 | 0.040341718 | 2 | 0.030105675 | 0 | 0 |
| 59 | SERPINE1 | 2 | 0.011904762 | 2 | 0 | 0 | 0 |
| 60 | PTGES | 124 | 0.039849977 | 2 | 0.012510073 | 0 | 0 |
| 61 | ACHE | 0 | 0.011903095 | 1 | 0 | 0 | 0 |
| 62 | ADIPOQ | 0 | 0.039152464 | 1 | 0.004477769 | 0 | 0 |
| 63 | HSPB1 | 0 | 0.040265277 | 1 | 0.03551425 | 0 | 0 |
| 64 | BACE1 | 0 | 0.011903095 | 1 | 0 | 0 | 0 |
| 65 | SOD1 | 0 | 0.012043072 | 1 | 0 | 0 | 0 |
| 66 | CD40LG | 0 | 0.039406583 | 1 | 0.009945426 | 0 | 0 |
| 67 | SELE | 0 | 0.039887377 | 1 | 0.008687723 | 0 | 0 |
| 68 | MMP2 | 0 | 0.011903095 | 1 | 0 | 0 | 0 |
| 69 | COL3A1 | 0 | 0.011903095 | 1 | 0 | 0 | 0 |
| 70 | CXCL11 | 0 | 0.039188566 | 1 | 0.006897212 | 0 | 0 |
| 71 | SULT1E1 | 0 | 0.039044557 | 1 | 0.006025582 | 0 | 0 |
| 72 | DPP4 | 0 | 0.038937242 | 1 | 0.002115443 | 0 | 0 |
| 73 | F3 | 0 | 0.011764706 | 1 | 0 | 0 | 0 |
| 74 | F7 | 0 | 0.011764706 | 1 | 0 | 0 | 0 |
| 75 | GOT1 | 0 | 0.011764706 | 1 | 0 | 0 | 0 |
| 76 | TYR | 0 | 0.011764706 | 1 | 0 | 0 | 0 |
| 77 | NFE2L2 | 0 | 0.012043072 | 1 | 0 | 0 | 0 |
| 78 | IGF2 | 0 | 0.011764706 | 1 | 0 | 0 | 0 |
| 79 | IGFBP3 | 0 | 0.011764706 | 1 | 0 | 0 | 0 |
| 80 | MMP1 | 0 | 0.038919414 | 1 | 0.001864039 | 0 | 0 |
| 81 | NCF1 | 0 | 0.039279113 | 1 | 0.005486221 | 0 | 0 |
| 82 | PPP3CA | 0 | 0.039188566 | 1 | 0.00453352 | 0 | 0 |
| 83 | NOS2 | 0 | 0.040265277 | 1 | 0.029203398 | 0 | 0 |
| 84 | PLAT | 0 | 0.011903095 | 1 | 0 | 0 | 0 |
| 85 | PLAU | 0 | 0.011903095 | 1 | 0 | 0 | 0 |
| 86 | PTGS1 | 0 | 0.038724374 | 1 | 0.001876703 | 0 | 0 |

**Supplementary Table 7.** Nine hundred and sixty-five biological processes, cellular components, and molecular functions were enriched based on GO analysis

| Category | GO | Description |
| --- | --- | --- |
| GO Molecular Functions | GO:0005126 | cytokine receptor binding |
| GO Molecular Functions | GO:0005125 | cytokine activity |
| GO Molecular Functions | GO:0005102 | signaling receptor binding |
| GO Molecular Functions | GO:0046983 | protein dimerization activity |
| GO Molecular Functions | GO:0042802 | identical protein binding |
| GO Molecular Functions | GO:0019899 | enzyme binding |
| GO Molecular Functions | GO:0004175 | endopeptidase activity |
| GO Molecular Functions | GO:0008233 | peptidase activity |
| GO Molecular Functions | GO:0042803 | protein homodimerization activity |
| GO Molecular Functions | GO:0004712 | protein serine/threonine/tyrosine kinase activity |
| GO Molecular Functions | GO:0048018 | receptor ligand activity |
| GO Molecular Functions | GO:0030546 | signaling receptor activator activity |
| GO Molecular Functions | GO:0030545 | signaling receptor regulator activity |
| GO Molecular Functions | GO:0070851 | growth factor receptor binding |
| GO Molecular Functions | GO:0042379 | chemokine receptor binding |
| GO Molecular Functions | GO:0004601 | peroxidase activity |
| GO Molecular Functions | GO:0008237 | metallopeptidase activity |
| GO Molecular Functions | GO:0020037 | heme binding |
| GO Molecular Functions | GO:0004672 | protein kinase activity |
| GO Molecular Functions | GO:0002020 | protease binding |
| GO Molecular Functions | GO:0046982 | protein heterodimerization activity |
| GO Molecular Functions | GO:0001540 | amyloid-beta binding |
| GO Molecular Functions | GO:0004222 | metalloendopeptidase activity |
| GO Molecular Functions | GO:0106310 | protein serine kinase activity |
| GO Molecular Functions | GO:0038023 | signaling receptor activity |
| GO Molecular Functions | GO:0060089 | molecular transducer activity |
| GO Molecular Functions | GO:0015108 | chloride transmembrane transporter activity |
| GO Molecular Functions | GO:0016684 | oxidoreductase activity, acting on peroxide as acceptor |
| GO Molecular Functions | GO:0050661 | NADP binding |
| GO Molecular Functions | GO:0016209 | antioxidant activity |
| GO Molecular Functions | GO:0046906 | tetrapyrrole binding |
| GO Molecular Functions | GO:0098772 | molecular function regulator activity |
| GO Molecular Functions | GO:0004674 | protein serine/threonine kinase activity |
| GO Molecular Functions | GO:0015459 | potassium channel regulator activity |
| GO Molecular Functions | GO:0016773 | phosphotransferase activity, alcohol group as acceptor |
| GO Molecular Functions | GO:0008270 | zinc ion binding |
| GO Molecular Functions | GO:1901682 | sulfur compound transmembrane transporter activity |
| GO Molecular Functions | GO:0004888 | transmembrane signaling receptor activity |
| GO Molecular Functions | GO:0001221 | transcription coregulator binding |
| GO Molecular Functions | GO:0015370 | solute:sodium symporter activity |
| GO Molecular Functions | GO:0042626 | ATPase-coupled transmembrane transporter activity |
| GO Molecular Functions | GO:0046914 | transition metal ion binding |
| GO Molecular Functions | GO:0004252 | serine-type endopeptidase activity |
| GO Molecular Functions | GO:0008509 | anion transmembrane transporter activity |
| GO Molecular Functions | GO:1990782 | protein tyrosine kinase binding |
| GO Molecular Functions | GO:0000976 | transcription cis-regulatory region binding |
| GO Molecular Functions | GO:0001067 | transcription regulatory region nucleic acid binding |
| GO Molecular Functions | GO:0043565 | sequence-specific DNA binding |
| GO Molecular Functions | GO:0000978 | RNA polymerase II cis-regulatory region sequence-specific DNA binding |
| GO Molecular Functions | GO:0003690 | double-stranded DNA binding |
| GO Molecular Functions | GO:0004497 | monooxygenase activity |
| GO Molecular Functions | GO:0016301 | kinase activity |
| GO Molecular Functions | GO:0008236 | serine-type peptidase activity |
| GO Molecular Functions | GO:0016705 | oxidoreductase activity, acting on paired donors, with incorporation or reduction of molecular oxygen |
| GO Molecular Functions | GO:0015103 | inorganic anion transmembrane transporter activity |
| GO Molecular Functions | GO:0017171 | serine hydrolase activity |
| GO Molecular Functions | GO:0015294 | solute:cation symporter activity |
| GO Molecular Functions | GO:1990837 | sequence-specific double-stranded DNA binding |
| GO Molecular Functions | GO:0003700 | DNA-binding transcription factor activity |
| GO Molecular Functions | GO:0008201 | heparin binding |
| GO Molecular Functions | GO:0042277 | peptide binding |
| GO Molecular Functions | GO:0030594 | neurotransmitter receptor activity |
| GO Molecular Functions | GO:0000987 | cis-regulatory region sequence-specific DNA binding |
| GO Molecular Functions | GO:0000977 | RNA polymerase II transcription regulatory region sequence-specific DNA binding |
| GO Molecular Functions | GO:0005496 | steroid binding |
| GO Molecular Functions | GO:0072341 | modified amino acid binding |
| GO Cellular Components | GO:0005615 | extracellular space |
| GO Cellular Components | GO:0045121 | membrane raft |
| GO Cellular Components | GO:0098857 | membrane microdomain |
| GO Cellular Components | GO:0000307 | cyclin-dependent protein kinase holoenzyme complex |
| GO Cellular Components | GO:0098590 | plasma membrane region |
| GO Cellular Components | GO:0009986 | cell surface |
| GO Cellular Components | GO:0005901 | caveola |
| GO Cellular Components | GO:0044853 | plasma membrane raft |
| GO Cellular Components | GO:1902554 | serine/threonine protein kinase complex |
| GO Cellular Components | GO:1902911 | protein kinase complex |
| GO Cellular Components | GO:0045177 | apical part of cell |
| GO Cellular Components | GO:0016324 | apical plasma membrane |
| GO Cellular Components | GO:0017053 | transcription repressor complex |
| GO Biological Processes | GO:0048584 | positive regulation of response to stimulus |
| GO Biological Processes | GO:0071310 | cellular response to organic substance |
| GO Biological Processes | GO:1901701 | cellular response to oxygen-containing compound |
| GO Biological Processes | GO:1901700 | response to oxygen-containing compound |
| GO Biological Processes | GO:0042981 | regulation of apoptotic process |
| GO Biological Processes | GO:0006915 | apoptotic process |
| GO Biological Processes | GO:0043067 | regulation of programmed cell death |
| GO Biological Processes | GO:0012501 | programmed cell death |
| GO Biological Processes | GO:0042127 | regulation of cell population proliferation |
| GO Biological Processes | GO:0051240 | positive regulation of multicellular organismal process |
| GO Biological Processes | GO:0050793 | regulation of developmental process |
| GO Biological Processes | GO:0010941 | regulation of cell death |
| GO Biological Processes | GO:0009719 | response to endogenous stimulus |
| GO Biological Processes | GO:0010628 | positive regulation of gene expression |
| GO Biological Processes | GO:0008219 | cell death |
| GO Biological Processes | GO:0040012 | regulation of locomotion |
| GO Biological Processes | GO:0008283 | cell population proliferation |
| GO Biological Processes | GO:0030334 | regulation of cell migration |
| GO Biological Processes | GO:0043085 | positive regulation of catalytic activity |
| GO Biological Processes | GO:1901698 | response to nitrogen compound |
| GO Biological Processes | GO:0048878 | chemical homeostasis |
| GO Biological Processes | GO:0006468 | protein phosphorylation |
| GO Biological Processes | GO:2000026 | regulation of multicellular organismal development |
| GO Biological Processes | GO:2000145 | regulation of cell motility |
| GO Biological Processes | GO:0032101 | regulation of response to external stimulus |
| GO Biological Processes | GO:0044093 | positive regulation of molecular function |
| GO Biological Processes | GO:1901652 | response to peptide |
| GO Biological Processes | GO:0042592 | homeostatic process |
| GO Biological Processes | GO:0051270 | regulation of cellular component movement |
| GO Biological Processes | GO:0043066 | negative regulation of apoptotic process |
| GO Biological Processes | GO:1901699 | cellular response to nitrogen compound |
| GO Biological Processes | GO:0010647 | positive regulation of cell communication |
| GO Biological Processes | GO:0043069 | negative regulation of programmed cell death |
| GO Biological Processes | GO:0016310 | phosphorylation |
| GO Biological Processes | GO:0023056 | positive regulation of signaling |
| GO Biological Processes | GO:0010243 | response to organonitrogen compound |
| GO Biological Processes | GO:0016477 | cell migration |
| GO Biological Processes | GO:0050878 | regulation of body fluid levels |
| GO Biological Processes | GO:0006954 | inflammatory response |
| GO Biological Processes | GO:0040017 | positive regulation of locomotion |
| GO Biological Processes | GO:0046903 | secretion |
| GO Biological Processes | GO:0007267 | cell-cell signaling |
| GO Biological Processes | GO:0006809 | nitric oxide biosynthetic process |
| GO Biological Processes | GO:0001819 | positive regulation of cytokine production |
| GO Biological Processes | GO:0023061 | signal release |
| GO Biological Processes | GO:0060548 | negative regulation of cell death |
| GO Biological Processes | GO:0071495 | cellular response to endogenous stimulus |
| GO Biological Processes | GO:0009967 | positive regulation of signal transduction |
| GO Biological Processes | GO:0001934 | positive regulation of protein phosphorylation |
| GO Biological Processes | GO:0019220 | regulation of phosphate metabolic process |
| GO Biological Processes | GO:0051174 | regulation of phosphorus metabolic process |
| GO Biological Processes | GO:0051247 | positive regulation of protein metabolic process |
| GO Biological Processes | GO:0008284 | positive regulation of cell population proliferation |
| GO Biological Processes | GO:1903530 | regulation of secretion by cell |
| GO Biological Processes | GO:0051046 | regulation of secretion |
| GO Biological Processes | GO:0001816 | cytokine production |
| GO Biological Processes | GO:0046209 | nitric oxide metabolic process |
| GO Biological Processes | GO:0140352 | export from cell |
| GO Biological Processes | GO:0009306 | protein secretion |
| GO Biological Processes | GO:0010562 | positive regulation of phosphorus metabolic process |
| GO Biological Processes | GO:0045937 | positive regulation of phosphate metabolic process |
| GO Biological Processes | GO:0035592 | establishment of protein localization to extracellular region |
| GO Biological Processes | GO:0035556 | intracellular signal transduction |
| GO Biological Processes | GO:0071692 | protein localization to extracellular region |
| GO Biological Processes | GO:0042327 | positive regulation of phosphorylation |
| GO Biological Processes | GO:2001057 | reactive nitrogen species metabolic process |
| GO Biological Processes | GO:0042325 | regulation of phosphorylation |
| GO Biological Processes | GO:0042886 | amide transport |
| GO Biological Processes | GO:0003008 | system process |
| GO Biological Processes | GO:0050708 | regulation of protein secretion |
| GO Biological Processes | GO:0034097 | response to cytokine |
| GO Biological Processes | GO:0071417 | cellular response to organonitrogen compound |
| GO Biological Processes | GO:0048870 | cell motility |
| GO Biological Processes | GO:0051674 | localization of cell |
| GO Biological Processes | GO:0009653 | anatomical structure morphogenesis |
| GO Biological Processes | GO:0030155 | regulation of cell adhesion |
| GO Biological Processes | GO:0000165 | MAPK cascade |
| GO Biological Processes | GO:0071345 | cellular response to cytokine stimulus |
| GO Biological Processes | GO:0030335 | positive regulation of cell migration |
| GO Biological Processes | GO:0032940 | secretion by cell |
| GO Biological Processes | GO:0051049 | regulation of transport |
| GO Biological Processes | GO:0001932 | regulation of protein phosphorylation |
| GO Biological Processes | GO:0001817 | regulation of cytokine production |
| GO Biological Processes | GO:0009628 | response to abiotic stimulus |
| GO Biological Processes | GO:0032270 | positive regulation of cellular protein metabolic process |
| GO Biological Processes | GO:0099536 | synaptic signaling |
| GO Biological Processes | GO:0045595 | regulation of cell differentiation |
| GO Biological Processes | GO:0031401 | positive regulation of protein modification process |
| GO Biological Processes | GO:0040011 | locomotion |
| GO Biological Processes | GO:2000147 | positive regulation of cell motility |
| GO Biological Processes | GO:1902533 | positive regulation of intracellular signal transduction |
| GO Biological Processes | GO:0009891 | positive regulation of biosynthetic process |
| GO Biological Processes | GO:0050790 | regulation of catalytic activity |
| GO Biological Processes | GO:0001775 | cell activation |
| GO Biological Processes | GO:0051272 | positive regulation of cellular component movement |
| GO Biological Processes | GO:0051241 | negative regulation of multicellular organismal process |
| GO Biological Processes | GO:0050920 | regulation of chemotaxis |
| GO Biological Processes | GO:0080134 | regulation of response to stress |
| GO Biological Processes | GO:0022407 | regulation of cell-cell adhesion |
| GO Biological Processes | GO:0033500 | carbohydrate homeostasis |
| GO Biological Processes | GO:0042593 | glucose homeostasis |
| GO Biological Processes | GO:0055082 | cellular chemical homeostasis |
| GO Biological Processes | GO:0031328 | positive regulation of cellular biosynthetic process |
| GO Biological Processes | GO:0032268 | regulation of cellular protein metabolic process |
| GO Biological Processes | GO:0048585 | negative regulation of response to stimulus |
| GO Biological Processes | GO:0001659 | temperature homeostasis |
| GO Biological Processes | GO:0019725 | cellular homeostasis |
| GO Biological Processes | GO:0006928 | movement of cell or subcellular component |
| GO Biological Processes | GO:0009725 | response to hormone |
| GO Biological Processes | GO:0032103 | positive regulation of response to external stimulus |
| GO Biological Processes | GO:0010573 | vascular endothelial growth factor production |
| GO Biological Processes | GO:1901653 | cellular response to peptide |
| GO Biological Processes | GO:0042060 | wound healing |
| GO Biological Processes | GO:0050673 | epithelial cell proliferation |
| GO Biological Processes | GO:0006357 | regulation of transcription by RNA polymerase II |
| GO Biological Processes | GO:0015833 | peptide transport |
| GO Biological Processes | GO:0009611 | response to wounding |
| GO Biological Processes | GO:0009890 | negative regulation of biosynthetic process |
| GO Biological Processes | GO:0031399 | regulation of protein modification process |
| GO Biological Processes | GO:0044057 | regulation of system process |
| GO Biological Processes | GO:1902531 | regulation of intracellular signal transduction |
| GO Biological Processes | GO:0071887 | leukocyte apoptotic process |
| GO Biological Processes | GO:0043434 | response to peptide hormone |
| GO Biological Processes | GO:0060326 | cell chemotaxis |
| GO Biological Processes | GO:0070661 | leukocyte proliferation |
| GO Biological Processes | GO:1903037 | regulation of leukocyte cell-cell adhesion |
| GO Biological Processes | GO:0003013 | circulatory system process |
| GO Biological Processes | GO:0043065 | positive regulation of apoptotic process |
| GO Biological Processes | GO:0051093 | negative regulation of developmental process |
| GO Biological Processes | GO:0045935 | positive regulation of nucleobase-containing compound metabolic process |
| GO Biological Processes | GO:0043068 | positive regulation of programmed cell death |
| GO Biological Processes | GO:0002682 | regulation of immune system process |
| GO Biological Processes | GO:0007186 | G protein-coupled receptor signaling pathway |
| GO Biological Processes | GO:0051094 | positive regulation of developmental process |
| GO Biological Processes | GO:0045428 | regulation of nitric oxide biosynthetic process |
| GO Biological Processes | GO:0050921 | positive regulation of chemotaxis |
| GO Biological Processes | GO:0071902 | positive regulation of protein serine/threonine kinase activity |
| GO Biological Processes | GO:0043408 | regulation of MAPK cascade |
| GO Biological Processes | GO:0071214 | cellular response to abiotic stimulus |
| GO Biological Processes | GO:0104004 | cellular response to environmental stimulus |
| GO Biological Processes | GO:0007167 | enzyme linked receptor protein signaling pathway |
| GO Biological Processes | GO:0071900 | regulation of protein serine/threonine kinase activity |
| GO Biological Processes | GO:0090087 | regulation of peptide transport |
| GO Biological Processes | GO:0099537 | trans-synaptic signaling |
| GO Biological Processes | GO:0045893 | positive regulation of transcription, DNA-templated |
| GO Biological Processes | GO:1903508 | positive regulation of nucleic acid-templated transcription |
| GO Biological Processes | GO:0007596 | blood coagulation |
| GO Biological Processes | GO:0007599 | hemostasis |
| GO Biological Processes | GO:1901214 | regulation of neuron death |
| GO Biological Processes | GO:0023057 | negative regulation of signaling |
| GO Biological Processes | GO:0018209 | peptidyl-serine modification |
| GO Biological Processes | GO:0050817 | coagulation |
| GO Biological Processes | GO:1902680 | positive regulation of RNA biosynthetic process |
| GO Biological Processes | GO:0045892 | negative regulation of transcription, DNA-templated |
| GO Biological Processes | GO:0006952 | defense response |
| GO Biological Processes | GO:1902679 | negative regulation of RNA biosynthetic process |
| GO Biological Processes | GO:1903507 | negative regulation of nucleic acid-templated transcription |
| GO Biological Processes | GO:0032943 | mononuclear cell proliferation |
| GO Biological Processes | GO:0046651 | lymphocyte proliferation |
| GO Biological Processes | GO:0062012 | regulation of small molecule metabolic process |
| GO Biological Processes | GO:0006690 | icosanoid metabolic process |
| GO Biological Processes | GO:0071356 | cellular response to tumor necrosis factor |
| GO Biological Processes | GO:0045931 | positive regulation of mitotic cell cycle |
| GO Biological Processes | GO:0006366 | transcription by RNA polymerase II |
| GO Biological Processes | GO:0010942 | positive regulation of cell death |
| GO Biological Processes | GO:0007159 | leukocyte cell-cell adhesion |
| GO Biological Processes | GO:0048514 | blood vessel morphogenesis |
| GO Biological Processes | GO:0098609 | cell-cell adhesion |
| GO Biological Processes | GO:0051253 | negative regulation of RNA metabolic process |
| GO Biological Processes | GO:0051128 | regulation of cellular component organization |
| GO Biological Processes | GO:0045944 | positive regulation of transcription by RNA polymerase II |
| GO Biological Processes | GO:0001568 | blood vessel development |
| GO Biological Processes | GO:0006508 | proteolysis |
| GO Biological Processes | GO:0007169 | transmembrane receptor protein tyrosine kinase signaling pathway |
| GO Biological Processes | GO:0051345 | positive regulation of hydrolase activity |
| GO Biological Processes | GO:0080164 | regulation of nitric oxide metabolic process |
| GO Biological Processes | GO:0007188 | adenylate cyclase-modulating G protein-coupled receptor signaling pathway |
| GO Biological Processes | GO:0010557 | positive regulation of macromolecule biosynthetic process |
| GO Biological Processes | GO:0007166 | cell surface receptor signaling pathway |
| GO Biological Processes | GO:0051172 | negative regulation of nitrogen compound metabolic process |
| GO Biological Processes | GO:0044839 | cell cycle G2/M phase transition |
| GO Biological Processes | GO:0010817 | regulation of hormone levels |
| GO Biological Processes | GO:0031324 | negative regulation of cellular metabolic process |
| GO Biological Processes | GO:0031327 | negative regulation of cellular biosynthetic process |
| GO Biological Processes | GO:0006953 | acute-phase response |
| GO Biological Processes | GO:0046879 | hormone secretion |
| GO Biological Processes | GO:0051052 | regulation of DNA metabolic process |
| GO Biological Processes | GO:0001944 | vasculature development |
| GO Biological Processes | GO:0034612 | response to tumor necrosis factor |
| GO Biological Processes | GO:0046883 | regulation of hormone secretion |
| GO Biological Processes | GO:0070997 | neuron death |
| GO Biological Processes | GO:0018105 | peptidyl-serine phosphorylation |
| GO Biological Processes | GO:0030595 | leukocyte chemotaxis |
| GO Biological Processes | GO:0097190 | apoptotic signaling pathway |
| GO Biological Processes | GO:0050727 | regulation of inflammatory response |
| GO Biological Processes | GO:0042110 | T cell activation |
| GO Biological Processes | GO:0010648 | negative regulation of cell communication |
| GO Biological Processes | GO:0045934 | negative regulation of nucleobase-containing compound metabolic process |
| GO Biological Processes | GO:0030162 | regulation of proteolysis |
| GO Biological Processes | GO:0070663 | regulation of leukocyte proliferation |
| GO Biological Processes | GO:1903039 | positive regulation of leukocyte cell-cell adhesion |
| GO Biological Processes | GO:0045787 | positive regulation of cell cycle |
| GO Biological Processes | GO:0042100 | B cell proliferation |
| GO Biological Processes | GO:0048871 | multicellular organismal homeostasis |
| GO Biological Processes | GO:0009914 | hormone transport |
| GO Biological Processes | GO:0099177 | regulation of trans-synaptic signaling |
| GO Biological Processes | GO:0002526 | acute inflammatory response |
| GO Biological Processes | GO:0030879 | mammary gland development |
| GO Biological Processes | GO:0006355 | regulation of transcription, DNA-templated |
| GO Biological Processes | GO:0051254 | positive regulation of RNA metabolic process |
| GO Biological Processes | GO:0050900 | leukocyte migration |
| GO Biological Processes | GO:0051051 | negative regulation of transport |
| GO Biological Processes | GO:1903506 | regulation of nucleic acid-templated transcription |
| GO Biological Processes | GO:0035239 | tube morphogenesis |
| GO Biological Processes | GO:0006935 | chemotaxis |
| GO Biological Processes | GO:0006979 | response to oxidative stress |
| GO Biological Processes | GO:0051338 | regulation of transferase activity |
| GO Biological Processes | GO:0007268 | chemical synaptic transmission |
| GO Biological Processes | GO:0098916 | anterograde trans-synaptic signaling |
| GO Biological Processes | GO:2001141 | regulation of RNA biosynthetic process |
| GO Biological Processes | GO:0030003 | cellular cation homeostasis |
| GO Biological Processes | GO:0042330 | taxis |
| GO Biological Processes | GO:0009887 | animal organ morphogenesis |
| GO Biological Processes | GO:0051223 | regulation of protein transport |
| GO Biological Processes | GO:0043523 | regulation of neuron apoptotic process |
| GO Biological Processes | GO:0045785 | positive regulation of cell adhesion |
| GO Biological Processes | GO:0071705 | nitrogen compound transport |
| GO Biological Processes | GO:0090257 | regulation of muscle system process |
| GO Biological Processes | GO:0045596 | negative regulation of cell differentiation |
| GO Biological Processes | GO:0034644 | cellular response to UV |
| GO Biological Processes | GO:0045766 | positive regulation of angiogenesis |
| GO Biological Processes | GO:0090276 | regulation of peptide hormone secretion |
| GO Biological Processes | GO:1904018 | positive regulation of vasculature development |
| GO Biological Processes | GO:0006873 | cellular ion homeostasis |
| GO Biological Processes | GO:0001836 | release of cytochrome c from mitochondria |
| GO Biological Processes | GO:0008015 | blood circulation |
| GO Biological Processes | GO:0050865 | regulation of cell activation |
| GO Biological Processes | GO:0031279 | regulation of cyclase activity |
| GO Biological Processes | GO:0002791 | regulation of peptide secretion |
| GO Biological Processes | GO:0022409 | positive regulation of cell-cell adhesion |
| GO Biological Processes | GO:0062197 | cellular response to chemical stress |
| GO Biological Processes | GO:0045321 | leukocyte activation |
| GO Biological Processes | GO:0045859 | regulation of protein kinase activity |
| GO Biological Processes | GO:0000278 | mitotic cell cycle |
| GO Biological Processes | GO:0006940 | regulation of smooth muscle contraction |
| GO Biological Processes | GO:0008285 | negative regulation of cell population proliferation |
| GO Biological Processes | GO:0030072 | peptide hormone secretion |
| GO Biological Processes | GO:0050731 | positive regulation of peptidyl-tyrosine phosphorylation |
| GO Biological Processes | GO:0051048 | negative regulation of secretion |
| GO Biological Processes | GO:0009894 | regulation of catabolic process |
| GO Biological Processes | GO:0046649 | lymphocyte activation |
| GO Biological Processes | GO:0002790 | peptide secretion |
| GO Biological Processes | GO:0042098 | T cell proliferation |
| GO Biological Processes | GO:0043270 | positive regulation of ion transport |
| GO Biological Processes | GO:0050870 | positive regulation of T cell activation |
| GO Biological Processes | GO:0071478 | cellular response to radiation |
| GO Biological Processes | GO:0018108 | peptidyl-tyrosine phosphorylation |
| GO Biological Processes | GO:0070201 | regulation of establishment of protein localization |
| GO Biological Processes | GO:0010524 | positive regulation of calcium ion transport into cytosol |
| GO Biological Processes | GO:0051349 | positive regulation of lyase activity |
| GO Biological Processes | GO:0002694 | regulation of leukocyte activation |
| GO Biological Processes | GO:0007346 | regulation of mitotic cell cycle |
| GO Biological Processes | GO:0071482 | cellular response to light stimulus |
| GO Biological Processes | GO:0035295 | tube development |
| GO Biological Processes | GO:0018212 | peptidyl-tyrosine modification |
| GO Biological Processes | GO:0009411 | response to UV |
| GO Biological Processes | GO:0000122 | negative regulation of transcription by RNA polymerase II |
| GO Biological Processes | GO:0010558 | negative regulation of macromolecule biosynthetic process |
| GO Biological Processes | GO:0032944 | regulation of mononuclear cell proliferation |
| GO Biological Processes | GO:0042113 | B cell activation |
| GO Biological Processes | GO:0050670 | regulation of lymphocyte proliferation |
| GO Biological Processes | GO:0051402 | neuron apoptotic process |
| GO Biological Processes | GO:0097529 | myeloid leukocyte migration |
| GO Biological Processes | GO:0000086 | G2/M transition of mitotic cell cycle |
| GO Biological Processes | GO:0048659 | smooth muscle cell proliferation |
| GO Biological Processes | GO:0048660 | regulation of smooth muscle cell proliferation |
| GO Biological Processes | GO:0050863 | regulation of T cell activation |
| GO Biological Processes | GO:0042509 | regulation of tyrosine phosphorylation of STAT protein |
| GO Biological Processes | GO:0051339 | regulation of lyase activity |
| GO Biological Processes | GO:0070227 | lymphocyte apoptotic process |
| GO Biological Processes | GO:1901992 | positive regulation of mitotic cell cycle phase transition |
| GO Biological Processes | GO:0030198 | extracellular matrix organization |
| GO Biological Processes | GO:0043281 | regulation of cysteine-type endopeptidase activity involved in apoptotic process |
| GO Biological Processes | GO:0045229 | external encapsulating structure organization |
| GO Biological Processes | GO:0072359 | circulatory system development |
| GO Biological Processes | GO:0030073 | insulin secretion |
| GO Biological Processes | GO:0034764 | positive regulation of transmembrane transport |
| GO Biological Processes | GO:0042176 | regulation of protein catabolic process |
| GO Biological Processes | GO:0043410 | positive regulation of MAPK cascade |
| GO Biological Processes | GO:0051336 | regulation of hydrolase activity |
| GO Biological Processes | GO:0007155 | cell adhesion |
| GO Biological Processes | GO:0051928 | positive regulation of calcium ion transport |
| GO Biological Processes | GO:0071466 | cellular response to xenobiotic stimulus |
| GO Biological Processes | GO:2000278 | regulation of DNA biosynthetic process |
| GO Biological Processes | GO:0043062 | extracellular structure organization |
| GO Biological Processes | GO:0050730 | regulation of peptidyl-tyrosine phosphorylation |
| GO Biological Processes | GO:0034762 | regulation of transmembrane transport |
| GO Biological Processes | GO:0051249 | regulation of lymphocyte activation |
| GO Biological Processes | GO:0010634 | positive regulation of epithelial cell migration |
| GO Biological Processes | GO:0051726 | regulation of cell cycle |
| GO Biological Processes | GO:0070848 | response to growth factor |
| GO Biological Processes | GO:0022603 | regulation of anatomical structure morphogenesis |
| GO Biological Processes | GO:0009416 | response to light stimulus |
| GO Biological Processes | GO:0071375 | cellular response to peptide hormone stimulus |
| GO Biological Processes | GO:0050678 | regulation of epithelial cell proliferation |
| GO Biological Processes | GO:1903047 | mitotic cell cycle process |
| GO Biological Processes | GO:0010605 | negative regulation of macromolecule metabolic process |
| GO Biological Processes | GO:0007200 | phospholipase C-activating G protein-coupled receptor signaling pathway |
| GO Biological Processes | GO:0007260 | tyrosine phosphorylation of STAT protein |
| GO Biological Processes | GO:0030888 | regulation of B cell proliferation |
| GO Biological Processes | GO:0050796 | regulation of insulin secretion |
| GO Biological Processes | GO:1903844 | regulation of cellular response to transforming growth factor beta stimulus |
| GO Biological Processes | GO:0003018 | vascular process in circulatory system |
| GO Biological Processes | GO:0050804 | modulation of chemical synaptic transmission |
| GO Biological Processes | GO:0051301 | cell division |
| GO Biological Processes | GO:0019221 | cytokine-mediated signaling pathway |
| GO Biological Processes | GO:2001233 | regulation of apoptotic signaling pathway |
| GO Biological Processes | GO:0055080 | cation homeostasis |
| GO Biological Processes | GO:0002688 | regulation of leukocyte chemotaxis |
| GO Biological Processes | GO:0006692 | prostanoid metabolic process |
| GO Biological Processes | GO:0006693 | prostaglandin metabolic process |
| GO Biological Processes | GO:0007189 | adenylate cyclase-activating G protein-coupled receptor signaling pathway |
| GO Biological Processes | GO:0030168 | platelet activation |
| GO Biological Processes | GO:0044772 | mitotic cell cycle phase transition |
| GO Biological Processes | GO:0007595 | lactation |
| GO Biological Processes | GO:0032757 | positive regulation of interleukin-8 production |
| GO Biological Processes | GO:0048708 | astrocyte differentiation |
| GO Biological Processes | GO:0090199 | regulation of release of cytochrome c from mitochondria |
| GO Biological Processes | GO:0120162 | positive regulation of cold-induced thermogenesis |
| GO Biological Processes | GO:0032870 | cellular response to hormone stimulus |
| GO Biological Processes | GO:0051347 | positive regulation of transferase activity |
| GO Biological Processes | GO:0042063 | gliogenesis |
| GO Biological Processes | GO:0050679 | positive regulation of epithelial cell proliferation |
| GO Biological Processes | GO:0070665 | positive regulation of leukocyte proliferation |
| GO Biological Processes | GO:0098771 | inorganic ion homeostasis |
| GO Biological Processes | GO:0002683 | negative regulation of immune system process |
| GO Biological Processes | GO:0048732 | gland development |
| GO Biological Processes | GO:0002521 | leukocyte differentiation |
| GO Biological Processes | GO:0002761 | regulation of myeloid leukocyte differentiation |
| GO Biological Processes | GO:0007589 | body fluid secretion |
| GO Biological Processes | GO:0050864 | regulation of B cell activation |
| GO Biological Processes | GO:0010038 | response to metal ion |
| GO Biological Processes | GO:0009314 | response to radiation |
| GO Biological Processes | GO:0045862 | positive regulation of proteolysis |
| GO Biological Processes | GO:0019216 | regulation of lipid metabolic process |
| GO Biological Processes | GO:0045597 | positive regulation of cell differentiation |
| GO Biological Processes | GO:0009410 | response to xenobiotic stimulus |
| GO Biological Processes | GO:0043549 | regulation of kinase activity |
| GO Biological Processes | GO:0010035 | response to inorganic substance |
| GO Biological Processes | GO:0051091 | positive regulation of DNA-binding transcription factor activity |
| GO Biological Processes | GO:2000116 | regulation of cysteine-type endopeptidase activity |
| GO Biological Processes | GO:0032637 | interleukin-8 production |
| GO Biological Processes | GO:0032677 | regulation of interleukin-8 production |
| GO Biological Processes | GO:0046456 | icosanoid biosynthetic process |
| GO Biological Processes | GO:2000106 | regulation of leukocyte apoptotic process |
| GO Biological Processes | GO:0050801 | ion homeostasis |
| GO Biological Processes | GO:0001936 | regulation of endothelial cell proliferation |
| GO Biological Processes | GO:0051781 | positive regulation of cell division |
| GO Biological Processes | GO:1903531 | negative regulation of secretion by cell |
| GO Biological Processes | GO:0051050 | positive regulation of transport |
| GO Biological Processes | GO:0006937 | regulation of muscle contraction |
| GO Biological Processes | GO:0048771 | tissue remodeling |
| GO Biological Processes | GO:0043254 | regulation of protein-containing complex assembly |
| GO Biological Processes | GO:0009968 | negative regulation of signal transduction |
| GO Biological Processes | GO:0048646 | anatomical structure formation involved in morphogenesis |
| GO Biological Processes | GO:0051129 | negative regulation of cellular component organization |
| GO Biological Processes | GO:0051130 | positive regulation of cellular component organization |
| GO Biological Processes | GO:0031347 | regulation of defense response |
| GO Biological Processes | GO:1902105 | regulation of leukocyte differentiation |
| GO Biological Processes | GO:0006939 | smooth muscle contraction |
| GO Biological Processes | GO:0006811 | ion transport |
| GO Biological Processes | GO:1902749 | regulation of cell cycle G2/M phase transition |
| GO Biological Processes | GO:0045860 | positive regulation of protein kinase activity |
| GO Biological Processes | GO:0045765 | regulation of angiogenesis |
| GO Biological Processes | GO:0051251 | positive regulation of lymphocyte activation |
| GO Biological Processes | GO:0002285 | lymphocyte activation involved in immune response |
| GO Biological Processes | GO:0032868 | response to insulin |
| GO Biological Processes | GO:0043269 | regulation of ion transport |
| GO Biological Processes | GO:0060429 | epithelium development |
| GO Biological Processes | GO:0050709 | negative regulation of protein secretion |
| GO Biological Processes | GO:1901989 | positive regulation of cell cycle phase transition |
| GO Biological Processes | GO:1901342 | regulation of vasculature development |
| GO Biological Processes | GO:1903706 | regulation of hemopoiesis |
| GO Biological Processes | GO:0050918 | positive chemotaxis |
| GO Biological Processes | GO:0051054 | positive regulation of DNA metabolic process |
| GO Biological Processes | GO:0090068 | positive regulation of cell cycle process |
| GO Biological Processes | GO:0034599 | cellular response to oxidative stress |
| GO Biological Processes | GO:0014070 | response to organic cyclic compound |
| GO Biological Processes | GO:0001938 | positive regulation of endothelial cell proliferation |
| GO Biological Processes | GO:0002532 | production of molecular mediator involved in inflammatory response |
| GO Biological Processes | GO:0022612 | gland morphogenesis |
| GO Biological Processes | GO:1901216 | positive regulation of neuron death |
| GO Biological Processes | GO:0019752 | carboxylic acid metabolic process |
| GO Biological Processes | GO:0007610 | behavior |
| GO Biological Processes | GO:0043436 | oxoacid metabolic process |
| GO Biological Processes | GO:0030855 | epithelial cell differentiation |
| GO Biological Processes | GO:0002685 | regulation of leukocyte migration |
| GO Biological Processes | GO:0045637 | regulation of myeloid cell differentiation |
| GO Biological Processes | GO:2001242 | regulation of intrinsic apoptotic signaling pathway |
| GO Biological Processes | GO:0002696 | positive regulation of leukocyte activation |
| GO Biological Processes | GO:0010876 | lipid localization |
| GO Biological Processes | GO:0033002 | muscle cell proliferation |
| GO Biological Processes | GO:0071453 | cellular response to oxygen levels |
| GO Biological Processes | GO:0032880 | regulation of protein localization |
| GO Biological Processes | GO:0001666 | response to hypoxia |
| GO Biological Processes | GO:1901990 | regulation of mitotic cell cycle phase transition |
| GO Biological Processes | GO:0019233 | sensory perception of pain |
| GO Biological Processes | GO:0032091 | negative regulation of protein binding |
| GO Biological Processes | GO:0006869 | lipid transport |
| GO Biological Processes | GO:0006875 | cellular metal ion homeostasis |
| GO Biological Processes | GO:0050867 | positive regulation of cell activation |
| GO Biological Processes | GO:0043405 | regulation of MAP kinase activity |
| GO Biological Processes | GO:0006082 | organic acid metabolic process |
| GO Biological Processes | GO:0036293 | response to decreased oxygen levels |
| GO Biological Processes | GO:0051480 | regulation of cytosolic calcium ion concentration |
| GO Biological Processes | GO:1903522 | regulation of blood circulation |
| GO Biological Processes | GO:0001525 | angiogenesis |
| GO Biological Processes | GO:0033554 | cellular response to stress |
| GO Biological Processes | GO:0060322 | head development |
| GO Biological Processes | GO:0050871 | positive regulation of B cell activation |
| GO Biological Processes | GO:1902106 | negative regulation of leukocyte differentiation |
| GO Biological Processes | GO:0001505 | regulation of neurotransmitter levels |
| GO Biological Processes | GO:0033559 | unsaturated fatty acid metabolic process |
| GO Biological Processes | GO:0051224 | negative regulation of protein transport |
| GO Biological Processes | GO:0048545 | response to steroid hormone |
| GO Biological Processes | GO:0007049 | cell cycle |
| GO Biological Processes | GO:0033674 | positive regulation of kinase activity |
| GO Biological Processes | GO:0010332 | response to gamma radiation |
| GO Biological Processes | GO:0030890 | positive regulation of B cell proliferation |
| GO Biological Processes | GO:0070228 | regulation of lymphocyte apoptotic process |
| GO Biological Processes | GO:2000134 | negative regulation of G1/S transition of mitotic cell cycle |
| GO Biological Processes | GO:0002684 | positive regulation of immune system process |
| GO Biological Processes | GO:0003012 | muscle system process |
| GO Biological Processes | GO:1904062 | regulation of cation transmembrane transport |
| GO Biological Processes | GO:0001935 | endothelial cell proliferation |
| GO Biological Processes | GO:0032946 | positive regulation of mononuclear cell proliferation |
| GO Biological Processes | GO:0044843 | cell cycle G1/S phase transition |
| GO Biological Processes | GO:0050671 | positive regulation of lymphocyte proliferation |
| GO Biological Processes | GO:1904950 | negative regulation of establishment of protein localization |
| GO Biological Processes | GO:0009888 | tissue development |
| GO Biological Processes | GO:0070555 | response to interleukin-1 |
| GO Biological Processes | GO:0071456 | cellular response to hypoxia |
| GO Biological Processes | GO:1903707 | negative regulation of hemopoiesis |
| GO Biological Processes | GO:0007249 | I-kappaB kinase/NF-kappaB signaling |
| GO Biological Processes | GO:0071216 | cellular response to biotic stimulus |
| GO Biological Processes | GO:1902532 | negative regulation of intracellular signal transduction |
| GO Biological Processes | GO:0048534 | hematopoietic or lymphoid organ development |
| GO Biological Processes | GO:0015031 | protein transport |
| GO Biological Processes | GO:0001503 | ossification |
| GO Biological Processes | GO:0001667 | ameboidal-type cell migration |
| GO Biological Processes | GO:0044770 | cell cycle phase transition |
| GO Biological Processes | GO:0010952 | positive regulation of peptidase activity |
| GO Biological Processes | GO:0042129 | regulation of T cell proliferation |
| GO Biological Processes | GO:0071674 | mononuclear cell migration |
| GO Biological Processes | GO:0006631 | fatty acid metabolic process |
| GO Biological Processes | GO:0031329 | regulation of cellular catabolic process |
| GO Biological Processes | GO:0006006 | glucose metabolic process |
| GO Biological Processes | GO:0030336 | negative regulation of cell migration |
| GO Biological Processes | GO:0010522 | regulation of calcium ion transport into cytosol |
| GO Biological Processes | GO:0017015 | regulation of transforming growth factor beta receptor signaling pathway |
| GO Biological Processes | GO:0030004 | cellular monovalent inorganic cation homeostasis |
| GO Biological Processes | GO:0033138 | positive regulation of peptidyl-serine phosphorylation |
| GO Biological Processes | GO:0036294 | cellular response to decreased oxygen levels |
| GO Biological Processes | GO:2001244 | positive regulation of intrinsic apoptotic signaling pathway |
| GO Biological Processes | GO:0052547 | regulation of peptidase activity |
| GO Biological Processes | GO:0033619 | membrane protein proteolysis |
| GO Biological Processes | GO:0035773 | insulin secretion involved in cellular response to glucose stimulus |
| GO Biological Processes | GO:0042743 | hydrogen peroxide metabolic process |
| GO Biological Processes | GO:0042987 | amyloid precursor protein catabolic process |
| GO Biological Processes | GO:0043525 | positive regulation of neuron apoptotic process |
| GO Biological Processes | GO:0046888 | negative regulation of hormone secretion |
| GO Biological Processes | GO:0050435 | amyloid-beta metabolic process |
| GO Biological Processes | GO:0150076 | neuroinflammatory response |
| GO Biological Processes | GO:2000300 | regulation of synaptic vesicle exocytosis |
| GO Biological Processes | GO:0008637 | apoptotic mitochondrial changes |
| GO Biological Processes | GO:0050729 | positive regulation of inflammatory response |
| GO Biological Processes | GO:0051302 | regulation of cell division |
| GO Biological Processes | GO:0033993 | response to lipid |
| GO Biological Processes | GO:0006629 | lipid metabolic process |
| GO Biological Processes | GO:0071363 | cellular response to growth factor stimulus |
| GO Biological Processes | GO:2000146 | negative regulation of cell motility |
| GO Biological Processes | GO:0002700 | regulation of production of molecular mediator of immune response |
| GO Biological Processes | GO:0015850 | organic hydroxy compound transport |
| GO Biological Processes | GO:0035150 | regulation of tube size |
| GO Biological Processes | GO:0035296 | regulation of tube diameter |
| GO Biological Processes | GO:0097191 | extrinsic apoptotic signaling pathway |
| GO Biological Processes | GO:0097746 | blood vessel diameter maintenance |
| GO Biological Processes | GO:0001678 | cellular glucose homeostasis |
| GO Biological Processes | GO:0002286 | T cell activation involved in immune response |
| GO Biological Processes | GO:0010595 | positive regulation of endothelial cell migration |
| GO Biological Processes | GO:0106106 | cold-induced thermogenesis |
| GO Biological Processes | GO:0120161 | regulation of cold-induced thermogenesis |
| GO Biological Processes | GO:1904064 | positive regulation of cation transmembrane transport |
| GO Biological Processes | GO:0010631 | epithelial cell migration |
| GO Biological Processes | GO:0090132 | epithelium migration |
| GO Biological Processes | GO:0051649 | establishment of localization in cell |
| GO Biological Processes | GO:0016485 | protein processing |
| GO Biological Processes | GO:0040013 | negative regulation of locomotion |
| GO Biological Processes | GO:0051271 | negative regulation of cellular component movement |
| GO Biological Processes | GO:0044262 | cellular carbohydrate metabolic process |
| GO Biological Processes | GO:0002520 | immune system development |
| GO Biological Processes | GO:0007162 | negative regulation of cell adhesion |
| GO Biological Processes | GO:0002534 | cytokine production involved in inflammatory response |
| GO Biological Processes | GO:0015844 | monoamine transport |
| GO Biological Processes | GO:0019229 | regulation of vasoconstriction |
| GO Biological Processes | GO:0042982 | amyloid precursor protein metabolic process |
| GO Biological Processes | GO:0043618 | regulation of transcription from RNA polymerase II promoter in response to stress |
| GO Biological Processes | GO:0048661 | positive regulation of smooth muscle cell proliferation |
| GO Biological Processes | GO:0051384 | response to glucocorticoid |
| GO Biological Processes | GO:0060147 | regulation of post-transcriptional gene silencing |
| GO Biological Processes | GO:0060964 | regulation of miRNA-mediated gene silencing |
| GO Biological Processes | GO:0070293 | renal absorption |
| GO Biological Processes | GO:1900015 | regulation of cytokine production involved in inflammatory response |
| GO Biological Processes | GO:1900368 | regulation of post-transcriptional gene silencing by RNA |
| GO Biological Processes | GO:1902807 | negative regulation of cell cycle G1/S phase transition |
| GO Biological Processes | GO:0032963 | collagen metabolic process |
| GO Biological Processes | GO:0071675 | regulation of mononuclear cell migration |
| GO Biological Processes | GO:2001257 | regulation of cation channel activity |
| GO Biological Processes | GO:0070482 | response to oxygen levels |
| GO Biological Processes | GO:0060284 | regulation of cell development |
| GO Biological Processes | GO:0055065 | metal ion homeostasis |
| GO Biological Processes | GO:0000302 | response to reactive oxygen species |
| GO Biological Processes | GO:0045732 | positive regulation of protein catabolic process |
| GO Biological Processes | GO:0022402 | cell cycle process |
| GO Biological Processes | GO:0009636 | response to toxic substance |
| GO Biological Processes | GO:0010632 | regulation of epithelial cell migration |
| GO Biological Processes | GO:0009896 | positive regulation of catabolic process |
| GO Biological Processes | GO:0007259 | receptor signaling pathway via JAK-STAT |
| GO Biological Processes | GO:0032635 | interleukin-6 production |
| GO Biological Processes | GO:0032675 | regulation of interleukin-6 production |
| GO Biological Processes | GO:0043154 | negative regulation of cysteine-type endopeptidase activity involved in apoptotic process |
| GO Biological Processes | GO:0043524 | negative regulation of neuron apoptotic process |
| GO Biological Processes | GO:0090130 | tissue migration |
| GO Biological Processes | GO:0031334 | positive regulation of protein-containing complex assembly |
| GO Biological Processes | GO:0045834 | positive regulation of lipid metabolic process |
| GO Biological Processes | GO:0050767 | regulation of neurogenesis |
| GO Biological Processes | GO:0071897 | DNA biosynthetic process |
| GO Biological Processes | GO:0051348 | negative regulation of transferase activity |
| GO Biological Processes | GO:0001516 | prostaglandin biosynthetic process |
| GO Biological Processes | GO:0001570 | vasculogenesis |
| GO Biological Processes | GO:0002762 | negative regulation of myeloid leukocyte differentiation |
| GO Biological Processes | GO:0035196 | production of miRNAs involved in gene silencing by miRNA |
| GO Biological Processes | GO:0043407 | negative regulation of MAP kinase activity |
| GO Biological Processes | GO:0043620 | regulation of DNA-templated transcription in response to stress |
| GO Biological Processes | GO:0046457 | prostanoid biosynthetic process |
| GO Biological Processes | GO:0050795 | regulation of behavior |
| GO Biological Processes | GO:0060966 | regulation of gene silencing by RNA |
| GO Biological Processes | GO:0097300 | programmed necrotic cell death |
| GO Biological Processes | GO:1903350 | response to dopamine |
| GO Biological Processes | GO:1903351 | cellular response to dopamine |
| GO Biological Processes | GO:1905330 | regulation of morphogenesis of an epithelium |
| GO Biological Processes | GO:1903131 | mononuclear cell differentiation |
| GO Biological Processes | GO:0018193 | peptidyl-amino acid modification |
| GO Biological Processes | GO:0010001 | glial cell differentiation |
| GO Biological Processes | GO:0009749 | response to glucose |
| GO Biological Processes | GO:0042752 | regulation of circadian rhythm |
| GO Biological Processes | GO:0097696 | receptor signaling pathway via STAT |
| GO Biological Processes | GO:0045184 | establishment of protein localization |
| GO Biological Processes | GO:0032787 | monocarboxylic acid metabolic process |
| GO Biological Processes | GO:0022411 | cellular component disassembly |
| GO Biological Processes | GO:0007399 | nervous system development |
| GO Biological Processes | GO:0007417 | central nervous system development |
| GO Biological Processes | GO:0006820 | anion transport |
| GO Biological Processes | GO:0010720 | positive regulation of cell development |
| GO Biological Processes | GO:0046890 | regulation of lipid biosynthetic process |
| GO Biological Processes | GO:0010389 | regulation of G2/M transition of mitotic cell cycle |
| GO Biological Processes | GO:0030512 | negative regulation of transforming growth factor beta receptor signaling pathway |
| GO Biological Processes | GO:0034103 | regulation of tissue remodeling |
| GO Biological Processes | GO:0046928 | regulation of neurotransmitter secretion |
| GO Biological Processes | GO:0048144 | fibroblast proliferation |
| GO Biological Processes | GO:0048145 | regulation of fibroblast proliferation |
| GO Biological Processes | GO:0050873 | brown fat cell differentiation |
| GO Biological Processes | GO:0070918 | primary sncRNA processing |
| GO Biological Processes | GO:0071867 | response to monoamine |
| GO Biological Processes | GO:0071868 | cellular response to monoamine stimulus |
| GO Biological Processes | GO:0071869 | response to catecholamine |
| GO Biological Processes | GO:0071870 | cellular response to catecholamine stimulus |
| GO Biological Processes | GO:1904427 | positive regulation of calcium ion transmembrane transport |
| GO Biological Processes | GO:0000082 | G1/S transition of mitotic cell cycle |
| GO Biological Processes | GO:0006275 | regulation of DNA replication |
| GO Biological Processes | GO:0007369 | gastrulation |
| GO Biological Processes | GO:0007565 | female pregnancy |
| GO Biological Processes | GO:0009746 | response to hexose |
| GO Biological Processes | GO:0033135 | regulation of peptidyl-serine phosphorylation |
| GO Biological Processes | GO:0034284 | response to monosaccharide |
| GO Biological Processes | GO:0034614 | cellular response to reactive oxygen species |
| GO Biological Processes | GO:0034767 | positive regulation of ion transmembrane transport |
| GO Biological Processes | GO:0042102 | positive regulation of T cell proliferation |
| GO Biological Processes | GO:0044703 | multi-organism reproductive process |
| GO Biological Processes | GO:0051100 | negative regulation of binding |
| GO Biological Processes | GO:0009895 | negative regulation of catabolic process |
| GO Biological Processes | GO:0019318 | hexose metabolic process |
| GO Biological Processes | GO:0002440 | production of molecular mediator of immune response |
| GO Biological Processes | GO:0006469 | negative regulation of protein kinase activity |
| GO Biological Processes | GO:0007204 | positive regulation of cytosolic calcium ion concentration |
| GO Biological Processes | GO:0042391 | regulation of membrane potential |
| GO Biological Processes | GO:0002702 | positive regulation of production of molecular mediator of immune response |
| GO Biological Processes | GO:0009743 | response to carbohydrate |
| GO Biological Processes | GO:0071621 | granulocyte chemotaxis |
| GO Biological Processes | GO:1990845 | adaptive thermogenesis |
| GO Biological Processes | GO:2000117 | negative regulation of cysteine-type endopeptidase activity |
| GO Biological Processes | GO:0071560 | cellular response to transforming growth factor beta stimulus |
| GO Biological Processes | GO:1903828 | negative regulation of protein localization |
| GO Biological Processes | GO:0006936 | muscle contraction |
| GO Biological Processes | GO:0001541 | ovarian follicle development |
| GO Biological Processes | GO:0002639 | positive regulation of immunoglobulin production |
| GO Biological Processes | GO:0006919 | activation of cysteine-type endopeptidase activity involved in apoptotic process |
| GO Biological Processes | GO:0032722 | positive regulation of chemokine production |
| GO Biological Processes | GO:0071346 | cellular response to interferon-gamma |
| GO Biological Processes | GO:0071347 | cellular response to interleukin-1 |
| GO Biological Processes | GO:1904892 | regulation of receptor signaling pathway via STAT |
| GO Biological Processes | GO:0031331 | positive regulation of cellular catabolic process |
| GO Biological Processes | GO:0048598 | embryonic morphogenesis |
| GO Biological Processes | GO:0044283 | small molecule biosynthetic process |
| GO Biological Processes | GO:0002573 | myeloid leukocyte differentiation |
| GO Biological Processes | GO:0051924 | regulation of calcium ion transport |
| GO Biological Processes | GO:0072593 | reactive oxygen species metabolic process |
| GO Biological Processes | GO:0002366 | leukocyte activation involved in immune response |
| GO Biological Processes | GO:0071706 | tumor necrosis factor superfamily cytokine production |
| GO Biological Processes | GO:1901991 | negative regulation of mitotic cell cycle phase transition |
| GO Biological Processes | GO:1903555 | regulation of tumor necrosis factor superfamily cytokine production |
| GO Biological Processes | GO:0010564 | regulation of cell cycle process |
| GO Biological Processes | GO:0034765 | regulation of ion transmembrane transport |
| GO Biological Processes | GO:0002263 | cell activation involved in immune response |
| GO Biological Processes | GO:0048511 | rhythmic process |
| GO Biological Processes | GO:0051604 | protein maturation |
| GO Biological Processes | GO:0046942 | carboxylic acid transport |
| GO Biological Processes | GO:0071559 | response to transforming growth factor beta |
| GO Biological Processes | GO:0051090 | regulation of DNA-binding transcription factor activity |
| GO Biological Processes | GO:0002287 | alpha-beta T cell activation involved in immune response |
| GO Biological Processes | GO:0002293 | alpha-beta T cell differentiation involved in immune response |
| GO Biological Processes | GO:0002294 | CD4-positive, alpha-beta T cell differentiation involved in immune response |
| GO Biological Processes | GO:0051053 | negative regulation of DNA metabolic process |
| GO Biological Processes | GO:0070265 | necrotic cell death |
| GO Biological Processes | GO:0090101 | negative regulation of transmembrane receptor protein serine/threonine kinase signaling pathway |
| GO Biological Processes | GO:0003014 | renal system process |
| GO Biological Processes | GO:0044706 | multi-multicellular organism process |
| GO Biological Processes | GO:0046631 | alpha-beta T cell activation |
| GO Biological Processes | GO:0060402 | calcium ion transport into cytosol |
| GO Biological Processes | GO:0005996 | monosaccharide metabolic process |
| GO Biological Processes | GO:1901987 | regulation of cell cycle phase transition |
| GO Biological Processes | GO:0044087 | regulation of cellular component biogenesis |
| GO Biological Processes | GO:0071407 | cellular response to organic cyclic compound |
| GO Biological Processes | GO:0006955 | immune response |
| GO Biological Processes | GO:0030098 | lymphocyte differentiation |
| GO Biological Processes | GO:0045165 | cell fate commitment |
| GO Biological Processes | GO:2001235 | positive regulation of apoptotic signaling pathway |
| GO Biological Processes | GO:0032409 | regulation of transporter activity |
| GO Biological Processes | GO:0033673 | negative regulation of kinase activity |
| GO Biological Processes | GO:0043393 | regulation of protein binding |
| GO Biological Processes | GO:0090287 | regulation of cellular response to growth factor stimulus |
| GO Biological Processes | GO:0048589 | developmental growth |
| GO Biological Processes | GO:0007423 | sensory organ development |
| GO Biological Processes | GO:0015718 | monocarboxylic acid transport |
| GO Biological Processes | GO:0016079 | synaptic vesicle exocytosis |
| GO Biological Processes | GO:0030641 | regulation of cellular pH |
| GO Biological Processes | GO:0034620 | cellular response to unfolded protein |
| GO Biological Processes | GO:0035637 | multicellular organismal signaling |
| GO Biological Processes | GO:0051279 | regulation of release of sequestered calcium ion into cytosol |
| GO Biological Processes | GO:0051588 | regulation of neurotransmitter transport |
| GO Biological Processes | GO:2000573 | positive regulation of DNA biosynthetic process |
| GO Biological Processes | GO:0080135 | regulation of cellular response to stress |
| GO Biological Processes | GO:0044092 | negative regulation of molecular function |
| GO Biological Processes | GO:0051248 | negative regulation of protein metabolic process |
| GO Biological Processes | GO:0060047 | heart contraction |
| GO Biological Processes | GO:0010959 | regulation of metal ion transport |
| GO Biological Processes | GO:0015711 | organic anion transport |
| GO Biological Processes | GO:0061041 | regulation of wound healing |
| GO Biological Processes | GO:0097193 | intrinsic apoptotic signaling pathway |
| GO Biological Processes | GO:0003015 | heart process |
| GO Biological Processes | GO:0045861 | negative regulation of proteolysis |
| GO Biological Processes | GO:0002292 | T cell differentiation involved in immune response |
| GO Biological Processes | GO:0002637 | regulation of immunoglobulin production |
| GO Biological Processes | GO:0002690 | positive regulation of leukocyte chemotaxis |
| GO Biological Processes | GO:0002695 | negative regulation of leukocyte activation |
| GO Biological Processes | GO:0031663 | lipopolysaccharide-mediated signaling pathway |
| GO Biological Processes | GO:0031960 | response to corticosteroid |
| GO Biological Processes | GO:0032602 | chemokine production |
| GO Biological Processes | GO:0032642 | regulation of chemokine production |
| GO Biological Processes | GO:0051341 | regulation of oxidoreductase activity |
| GO Biological Processes | GO:0071333 | cellular response to glucose stimulus |
| GO Biological Processes | GO:0006109 | regulation of carbohydrate metabolic process |
| GO Biological Processes | GO:0032869 | cellular response to insulin stimulus |
| GO Biological Processes | GO:0043409 | negative regulation of MAPK cascade |
| GO Biological Processes | GO:1901215 | negative regulation of neuron death |
| GO Biological Processes | GO:0048568 | embryonic organ development |
| GO Biological Processes | GO:0048608 | reproductive structure development |
| GO Biological Processes | GO:0061458 | reproductive system development |
| GO Biological Processes | GO:0044089 | positive regulation of cellular component biogenesis |
| GO Biological Processes | GO:0051960 | regulation of nervous system development |
| GO Biological Processes | GO:0005975 | carbohydrate metabolic process |
| GO Biological Processes | GO:0022008 | neurogenesis |
| GO Biological Processes | GO:0006836 | neurotransmitter transport |
| GO Biological Processes | GO:1903034 | regulation of response to wounding |
| GO Biological Processes | GO:1901565 | organonitrogen compound catabolic process |
| GO Biological Processes | GO:0008610 | lipid biosynthetic process |
| GO Biological Processes | GO:0015849 | organic acid transport |
| GO Biological Processes | GO:2001234 | negative regulation of apoptotic signaling pathway |
| GO Biological Processes | GO:0030097 | hemopoiesis |
| GO Biological Processes | GO:0007269 | neurotransmitter secretion |
| GO Biological Processes | GO:0034121 | regulation of toll-like receptor signaling pathway |
| GO Biological Processes | GO:0034341 | response to interferon-gamma |
| GO Biological Processes | GO:0034637 | cellular carbohydrate biosynthetic process |
| GO Biological Processes | GO:0034763 | negative regulation of transmembrane transport |
| GO Biological Processes | GO:0042310 | vasoconstriction |
| GO Biological Processes | GO:0045639 | positive regulation of myeloid cell differentiation |
| GO Biological Processes | GO:0071326 | cellular response to monosaccharide stimulus |
| GO Biological Processes | GO:0071331 | cellular response to hexose stimulus |
| GO Biological Processes | GO:0097006 | regulation of plasma lipoprotein particle levels |
| GO Biological Processes | GO:0099643 | signal release from synapse |
| GO Biological Processes | GO:0002697 | regulation of immune effector process |
| GO Biological Processes | GO:0071396 | cellular response to lipid |
| GO Biological Processes | GO:0032269 | negative regulation of cellular protein metabolic process |
| GO Biological Processes | GO:0002699 | positive regulation of immune effector process |
| GO Biological Processes | GO:0006898 | receptor-mediated endocytosis |
| GO Biological Processes | GO:0030217 | T cell differentiation |
| GO Biological Processes | GO:0070371 | ERK1 and ERK2 cascade |
| GO Biological Processes | GO:0001649 | osteoblast differentiation |
| GO Biological Processes | GO:0007179 | transforming growth factor beta receptor signaling pathway |
| GO Biological Processes | GO:0009266 | response to temperature stimulus |
| GO Biological Processes | GO:0060401 | cytosolic calcium ion transport |
| GO Biological Processes | GO:0097530 | granulocyte migration |
| GO Biological Processes | GO:0090066 | regulation of anatomical structure size |
| GO Biological Processes | GO:0044248 | cellular catabolic process |
| GO Biological Processes | GO:0006874 | cellular calcium ion homeostasis |
| GO Biological Processes | GO:0052548 | regulation of endopeptidase activity |
| GO Biological Processes | GO:0043542 | endothelial cell migration |
| GO Biological Processes | GO:0032102 | negative regulation of response to external stimulus |
| GO Biological Processes | GO:0051098 | regulation of binding |
| GO Biological Processes | GO:0006636 | unsaturated fatty acid biosynthetic process |
| GO Biological Processes | GO:0030593 | neutrophil chemotaxis |
| GO Biological Processes | GO:0050769 | positive regulation of neurogenesis |
| GO Biological Processes | GO:0071322 | cellular response to carbohydrate stimulus |
| GO Biological Processes | GO:1903557 | positive regulation of tumor necrosis factor superfamily cytokine production |
| GO Biological Processes | GO:2000045 | regulation of G1/S transition of mitotic cell cycle |
| GO Biological Processes | GO:0001818 | negative regulation of cytokine production |
| GO Biological Processes | GO:0071248 | cellular response to metal ion |
| GO Biological Processes | GO:0055074 | calcium ion homeostasis |
| GO Biological Processes | GO:0002237 | response to molecule of bacterial origin |
| GO Biological Processes | GO:0010506 | regulation of autophagy |
| GO Biological Processes | GO:0009790 | embryo development |
| GO Biological Processes | GO:0043086 | negative regulation of catalytic activity |
| GO Biological Processes | GO:1901615 | organic hydroxy compound metabolic process |
| GO Biological Processes | GO:0045930 | negative regulation of mitotic cell cycle |
| GO Biological Processes | GO:0090092 | regulation of transmembrane receptor protein serine/threonine kinase signaling pathway |
| GO Biological Processes | GO:0034504 | protein localization to nucleus |
| GO Biological Processes | GO:1901617 | organic hydroxy compound biosynthetic process |
| GO Biological Processes | GO:0002367 | cytokine production involved in immune response |
| GO Biological Processes | GO:0002718 | regulation of cytokine production involved in immune response |
| GO Biological Processes | GO:0016241 | regulation of macroautophagy |
| GO Biological Processes | GO:0035967 | cellular response to topologically incorrect protein |
| GO Biological Processes | GO:0043367 | CD4-positive, alpha-beta T cell differentiation |
| GO Biological Processes | GO:0071901 | negative regulation of protein serine/threonine kinase activity |
| GO Biological Processes | GO:0008202 | steroid metabolic process |
| GO Biological Processes | GO:0072503 | cellular divalent inorganic cation homeostasis |
| GO Biological Processes | GO:0008217 | regulation of blood pressure |
| GO Biological Processes | GO:0071222 | cellular response to lipopolysaccharide |
| GO Biological Processes | GO:0001654 | eye development |
| GO Biological Processes | GO:0006633 | fatty acid biosynthetic process |
| GO Biological Processes | GO:0010469 | regulation of signaling receptor activity |
| GO Biological Processes | GO:0019217 | regulation of fatty acid metabolic process |
| GO Biological Processes | GO:0022408 | negative regulation of cell-cell adhesion |
| GO Biological Processes | GO:0032755 | positive regulation of interleukin-6 production |
| GO Biological Processes | GO:0038127 | ERBB signaling pathway |
| GO Biological Processes | GO:0043280 | positive regulation of cysteine-type endopeptidase activity involved in apoptotic process |
| GO Biological Processes | GO:0045638 | negative regulation of myeloid cell differentiation |
| GO Biological Processes | GO:0050866 | negative regulation of cell activation |
| GO Biological Processes | GO:0051881 | regulation of mitochondrial membrane potential |
| GO Biological Processes | GO:1903305 | regulation of regulated secretory pathway |
| GO Biological Processes | GO:1904659 | glucose transmembrane transport |
| GO Biological Processes | GO:1905897 | regulation of response to endoplasmic reticulum stress |
| GO Biological Processes | GO:0055085 | transmembrane transport |
| GO Biological Processes | GO:0008016 | regulation of heart contraction |
| GO Biological Processes | GO:0008406 | gonad development |
| GO Biological Processes | GO:0008643 | carbohydrate transport |
| GO Biological Processes | GO:0043122 | regulation of I-kappaB kinase/NF-kappaB signaling |
| GO Biological Processes | GO:0045137 | development of primary sexual characteristics |
| GO Biological Processes | GO:0072507 | divalent inorganic cation homeostasis |
| GO Biological Processes | GO:0040007 | growth |
| GO Biological Processes | GO:0010629 | negative regulation of gene expression |
| GO Biological Processes | GO:0150063 | visual system development |
| GO Biological Processes | GO:2001020 | regulation of response to DNA damage stimulus |
| GO Biological Processes | GO:0030099 | myeloid cell differentiation |
| GO Biological Processes | GO:0032412 | regulation of ion transmembrane transporter activity |
| GO Biological Processes | GO:0045926 | negative regulation of growth |
| GO Biological Processes | GO:0055067 | monovalent inorganic cation homeostasis |
| GO Biological Processes | GO:0006606 | protein import into nucleus |
| GO Biological Processes | GO:0008645 | hexose transmembrane transport |
| GO Biological Processes | GO:0015749 | monosaccharide transmembrane transport |
| GO Biological Processes | GO:0019218 | regulation of steroid metabolic process |
| GO Biological Processes | GO:0021782 | glial cell development |
| GO Biological Processes | GO:0035195 | miRNA-mediated gene silencing |
| GO Biological Processes | GO:0051170 | import into nucleus |
| GO Biological Processes | GO:0062014 | negative regulation of small molecule metabolic process |
| GO Biological Processes | GO:0048880 | sensory system development |
| GO Biological Processes | GO:0015748 | organophosphate ester transport |
| GO Biological Processes | GO:0071241 | cellular response to inorganic substance |
| GO Biological Processes | GO:0002252 | immune effector process |
| GO Biological Processes | GO:0002377 | immunoglobulin production |
| GO Biological Processes | GO:0007093 | mitotic cell cycle checkpoint signaling |
| GO Biological Processes | GO:0008585 | female gonad development |
| GO Biological Processes | GO:0010906 | regulation of glucose metabolic process |
| GO Biological Processes | GO:0035194 | post-transcriptional gene silencing by RNA |
| GO Biological Processes | GO:0035710 | CD4-positive, alpha-beta T cell activation |
| GO Biological Processes | GO:0046545 | development of primary female sexual characteristics |
| GO Biological Processes | GO:0046632 | alpha-beta T cell differentiation |
| GO Biological Processes | GO:0046660 | female sex differentiation |
| GO Biological Processes | GO:0046889 | positive regulation of lipid biosynthetic process |
| GO Biological Processes | GO:0062207 | regulation of pattern recognition receptor signaling pathway |
| GO Biological Processes | GO:0072676 | lymphocyte migration |
| GO Biological Processes | GO:0006816 | calcium ion transport |
| GO Biological Processes | GO:0010563 | negative regulation of phosphorus metabolic process |
| GO Biological Processes | GO:0045936 | negative regulation of phosphate metabolic process |
| GO Biological Processes | GO:0071219 | cellular response to molecule of bacterial origin |
| GO Biological Processes | GO:1901988 | negative regulation of cell cycle phase transition |
| GO Biological Processes | GO:0006970 | response to osmotic stress |
| GO Biological Processes | GO:0007622 | rhythmic behavior |
| GO Biological Processes | GO:0010517 | regulation of phospholipase activity |
| GO Biological Processes | GO:0010939 | regulation of necrotic cell death |
| GO Biological Processes | GO:0014002 | astrocyte development |
| GO Biological Processes | GO:0014013 | regulation of gliogenesis |
| GO Biological Processes | GO:0016572 | histone phosphorylation |
| GO Biological Processes | GO:0022602 | ovulation cycle process |
| GO Biological Processes | GO:0030520 | intracellular estrogen receptor signaling pathway |
| GO Biological Processes | GO:0030574 | collagen catabolic process |
| GO Biological Processes | GO:0032370 | positive regulation of lipid transport |
| GO Biological Processes | GO:0034110 | regulation of homotypic cell-cell adhesion |
| GO Biological Processes | GO:0043903 | regulation of biological process involved in symbiotic interaction |
| GO Biological Processes | GO:0044070 | regulation of anion transport |
| GO Biological Processes | GO:0045740 | positive regulation of DNA replication |
| GO Biological Processes | GO:0046323 | glucose import |
| GO Biological Processes | GO:0048246 | macrophage chemotaxis |
| GO Biological Processes | GO:0048512 | circadian behavior |
| GO Biological Processes | GO:0048538 | thymus development |
| GO Biological Processes | GO:0048662 | negative regulation of smooth muscle cell proliferation |
| GO Biological Processes | GO:2000107 | negative regulation of leukocyte apoptotic process |
| GO Biological Processes | GO:0050877 | nervous system process |
| GO Biological Processes | GO:0010594 | regulation of endothelial cell migration |
| GO Biological Processes | GO:0016051 | carbohydrate biosynthetic process |
| GO Biological Processes | GO:0090596 | sensory organ morphogenesis |
| GO Biological Processes | GO:2000241 | regulation of reproductive process |
| GO Biological Processes | GO:0010822 | positive regulation of mitochondrion organization |
| GO Biological Processes | GO:0016441 | post-transcriptional gene silencing |
| GO Biological Processes | GO:0044264 | cellular polysaccharide metabolic process |
| GO Biological Processes | GO:0051209 | release of sequestered calcium ion into cytosol |
| GO Biological Processes | GO:1990266 | neutrophil migration |
| GO Biological Processes | GO:2001056 | positive regulation of cysteine-type endopeptidase activity |
| GO Biological Processes | GO:0060341 | regulation of cellular localization |
| GO Biological Processes | GO:0001933 | negative regulation of protein phosphorylation |
| GO Biological Processes | GO:0007623 | circadian rhythm |
| GO Biological Processes | GO:0010821 | regulation of mitochondrion organization |
| GO Biological Processes | GO:0098869 | cellular oxidant detoxification |
| GO Biological Processes | GO:0031349 | positive regulation of defense response |
| GO Biological Processes | GO:0000723 | telomere maintenance |
| GO Biological Processes | GO:0009408 | response to heat |
| GO Biological Processes | GO:0010675 | regulation of cellular carbohydrate metabolic process |
| GO Biological Processes | GO:0015914 | phospholipid transport |
| GO Biological Processes | GO:0030278 | regulation of ossification |
| GO Biological Processes | GO:0032200 | telomere organization |
| GO Biological Processes | GO:0044409 | entry into host |
| GO Biological Processes | GO:0051592 | response to calcium ion |
| GO Biological Processes | GO:0097553 | calcium ion transmembrane import into cytosol |
| GO Biological Processes | GO:1902806 | regulation of cell cycle G1/S phase transition |
| GO Biological Processes | GO:0001960 | negative regulation of cytokine-mediated signaling pathway |
| GO Biological Processes | GO:0006801 | superoxide metabolic process |
| GO Biological Processes | GO:0006984 | ER-nucleus signaling pathway |
| GO Biological Processes | GO:0010812 | negative regulation of cell-substrate adhesion |
| GO Biological Processes | GO:0032941 | secretion by tissue |
| GO Biological Processes | GO:0032964 | collagen biosynthetic process |
| GO Biological Processes | GO:0042149 | cellular response to glucose starvation |
| GO Biological Processes | GO:0043470 | regulation of carbohydrate catabolic process |
| GO Biological Processes | GO:0051937 | catecholamine transport |
| GO Biological Processes | GO:0060260 | regulation of transcription initiation from RNA polymerase II promoter |
| GO Biological Processes | GO:0061180 | mammary gland epithelium development |
| GO Biological Processes | GO:0061337 | cardiac conduction |
| GO Biological Processes | GO:1904994 | regulation of leukocyte adhesion to vascular endothelial cell |
| GO Biological Processes | GO:2000142 | regulation of DNA-templated transcription, initiation |
| GO Biological Processes | GO:0007548 | sex differentiation |
| GO Biological Processes | GO:0022898 | regulation of transmembrane transporter activity |
| GO Biological Processes | GO:0031330 | negative regulation of cellular catabolic process |
| GO Biological Processes | GO:0043491 | protein kinase B signaling |
| GO Biological Processes | GO:0048762 | mesenchymal cell differentiation |
| GO Biological Processes | GO:0051651 | maintenance of location in cell |
| GO Biological Processes | GO:0060627 | regulation of vesicle-mediated transport |
| GO Biological Processes | GO:0030163 | protein catabolic process |
| GO Biological Processes | GO:0050776 | regulation of immune response |

**Supplementary Table 8.** One hundred and sixty-six pathways were enriched by KEGG

| NO. | ID | Description | Count |
| --- | --- | --- | --- |
| 1 | bta04933 | AGE-RAGE signaling pathway in diabetic complications | 27 |
| 2 | bta05417 | Lipid and atherosclerosis | 32 |
| 3 | bta05418 | Fluid shear stress and atherosclerosis | 23 |
| 4 | bta05161 | Hepatitis B | 22 |
| 5 | bta05163 | Human cytomegalovirus infection | 25 |
| 6 | bta04657 | IL-17 signaling pathway | 17 |
| 7 | bta05205 | Proteoglycans in cancer | 22 |
| 8 | bta05164 | Influenza A | 20 |
| 9 | bta05160 | Hepatitis C | 19 |
| 10 | bta05222 | Small cell lung cancer | 15 |
| 11 | bta05167 | Kaposi sarcoma-associated herpesvirus infection | 21 |
| 12 | bta05219 | Bladder cancer | 11 |
| 13 | bta04218 | Cellular senescence | 18 |
| 14 | bta04668 | TNF signaling pathway | 16 |
| 15 | bta05142 | Chagas disease | 15 |
| 16 | bta04625 | C-type lectin receptor signaling pathway | 14 |
| 17 | bta04660 | T cell receptor signaling pathway | 14 |
| 18 | bta04151 | PI3K-Akt signaling pathway | 24 |
| 19 | bta05145 | Toxoplasmosis | 14 |
| 20 | bta05144 | Malaria | 11 |
| 21 | bta05169 | Epstein-Barr virus infection | 19 |
| 22 | bta05162 | Measles | 16 |
| 23 | bta05215 | Prostate cancer | 13 |
| 24 | bta04115 | p53 signaling pathway | 12 |
| 25 | bta04066 | HIF-1 signaling pathway | 13 |
| 26 | bta01522 | Endocrine resistance | 12 |
| 27 | bta05212 | Pancreatic cancer | 11 |
| 28 | bta04659 | Th17 cell differentiation | 13 |
| 29 | bta05146 | Amoebiasis | 13 |
| 30 | bta04210 | Apoptosis | 14 |
| 31 | bta04010 | MAPK signaling pathway | 19 |
| 32 | bta05152 | Tuberculosis | 16 |
| 33 | bta04068 | FoxO signaling pathway | 13 |
| 34 | bta04926 | Relaxin signaling pathway | 13 |
| 35 | bta05166 | Human T-cell leukemia virus 1 infection | 17 |
| 36 | bta05210 | Colorectal cancer | 11 |
| 37 | bta05321 | Inflammatory bowel disease | 10 |
| 38 | bta05223 | Non-small cell lung cancer | 10 |
| 39 | bta04620 | Toll-like receptor signaling pathway | 12 |
| 40 | bta04370 | VEGF signaling pathway | 9 |
| 41 | bta05220 | Chronic myeloid leukemia | 10 |
| 42 | bta05133 | Pertussis | 10 |
| 43 | bta05140 | Leishmaniasis | 10 |
| 44 | bta05143 | African trypanosomiasis | 8 |
| 45 | bta05206 | MicroRNAs in cancer | 18 |
| 46 | bta01521 | EGFR tyrosine kinase inhibitor resistance | 10 |
| 47 | bta04936 | Alcoholic liver disease | 13 |
| 48 | bta05323 | Rheumatoid arthritis | 11 |
| 49 | bta05135 | Yersinia infection | 13 |
| 50 | bta05207 | Chemical carcinogenesis - receptor activation | 15 |
| 51 | bta05221 | Acute myeloid leukemia | 9 |
| 52 | bta04064 | NF-kappa B signaling pathway | 11 |
| 53 | bta05165 | Human papillomavirus infection | 19 |
| 54 | bta04380 | Osteoclast differentiation | 12 |
| 55 | bta04915 | Estrogen signaling pathway | 12 |
| 56 | bta05235 | PD-L1 expression and PD-1 checkpoint pathway in cancer | 10 |
| 57 | bta04071 | Sphingolipid signaling pathway | 11 |
| 58 | bta05225 | Hepatocellular carcinoma | 13 |
| 59 | bta05170 | Human immunodeficiency virus 1 infection | 15 |
| 60 | bta04932 | Non-alcoholic fatty liver disease | 12 |
| 61 | bta05132 | Salmonella infection | 15 |
| 62 | bta05022 | Pathways of neurodegeneration - multiple diseases | 21 |
| 63 | bta01523 | Antifolate resistance | 7 |
| 64 | bta05208 | Chemical carcinogenesis - reactive oxygen species | 14 |
| 65 | bta04061 | Viral protein interaction with cytokine and cytokine receptor | 9 |
| 66 | bta04726 | Serotonergic synapse | 10 |
| 67 | bta05218 | Melanoma | 8 |
| 68 | bta04152 | AMPK signaling pathway | 10 |
| 69 | bta05020 | Prion disease | 15 |
| 70 | bta05214 | Glioma | 8 |
| 71 | bta04020 | Calcium signaling pathway | 14 |
| 72 | bta01524 | Platinum drug resistance | 8 |
| 73 | bta05010 | Alzheimer disease | 18 |
| 74 | bta04062 | Chemokine signaling pathway | 12 |
| 75 | bta05134 | Legionellosis | 7 |
| 76 | bta05213 | Endometrial cancer | 7 |
| 77 | bta05216 | Thyroid cancer | 6 |
| 78 | bta04917 | Prolactin signaling pathway | 8 |
| 79 | bta05202 | Transcriptional misregulation in cancer | 12 |
| 80 | bta04510 | Focal adhesion | 12 |
| 81 | bta04022 | cGMP-PKG signaling pathway | 11 |
| 82 | bta04914 | Progesterone-mediated oocyte maturation | 8 |
| 83 | bta04540 | Gap junction | 8 |
| 84 | bta05230 | Central carbon metabolism in cancer | 7 |
| 85 | bta05224 | Breast cancer | 10 |
| 86 | bta04919 | Thyroid hormone signaling pathway | 9 |
| 87 | bta05415 | Diabetic cardiomyopathy | 12 |
| 88 | bta00330 | Arginine and proline metabolism | 6 |
| 89 | bta05226 | Gastric cancer | 10 |
| 90 | bta04920 | Adipocytokine signaling pathway | 7 |
| 91 | bta04110 | Cell cycle | 9 |
| 92 | bta04658 | Th1 and Th2 cell differentiation | 8 |
| 93 | bta04621 | NOD-like receptor signaling pathway | 11 |
| 94 | bta04215 | Apoptosis - multiple species | 5 |
| 95 | bta04728 | Dopaminergic synapse | 9 |
| 96 | bta05330 | Allograft rejection | 6 |
| 97 | bta04931 | Insulin resistance | 8 |
| 98 | bta04371 | Apelin signaling pathway | 9 |
| 99 | bta04923 | Regulation of lipolysis in adipocytes | 6 |
| 100 | bta05012 | Parkinson disease | 13 |
| 101 | bta05171 | Coronavirus disease - COVID-19 | 13 |
| 102 | bta04024 | cAMP signaling pathway | 12 |
| 103 | bta04725 | Cholinergic synapse | 8 |
| 104 | bta04630 | JAK-STAT signaling pathway | 11 |
| 105 | bta04662 | B cell receptor signaling pathway | 7 |
| 106 | bta04217 | Necroptosis | 10 |
| 107 | bta04060 | Cytokine-cytokine receptor interaction | 14 |
| 108 | bta04929 | GnRH secretion | 6 |
| 109 | bta05332 | Graft-versus-host disease | 6 |
| 110 | bta04650 | Natural killer cell mediated cytotoxicity | 9 |
| 111 | bta05031 | Amphetamine addiction | 6 |
| 112 | bta04664 | Fc epsilon RI signaling pathway | 6 |
| 113 | bta04930 | Type II diabetes mellitus | 5 |
| 114 | bta05211 | Renal cell carcinoma | 6 |
| 115 | bta05030 | Cocaine addiction | 5 |
| 116 | bta04623 | Cytosolic DNA-sensing pathway | 6 |
| 117 | bta04910 | Insulin signaling pathway | 8 |
| 118 | bta05416 | Viral myocarditis | 6 |
| 119 | bta03320 | PPAR signaling pathway | 6 |
| 120 | bta04672 | Intestinal immune network for IgA production | 5 |
| 121 | bta04080 | Neuroactive ligand-receptor interaction | 14 |
| 122 | bta04921 | Oxytocin signaling pathway | 8 |
| 123 | bta04940 | Type I diabetes mellitus | 5 |
| 124 | bta04211 | Longevity regulating pathway | 6 |
| 125 | bta04722 | Neurotrophin signaling pathway | 7 |
| 126 | bta04610 | Complement and coagulation cascades | 6 |
| 127 | bta00350 | Tyrosine metabolism | 4 |
| 128 | bta05310 | Asthma | 4 |
| 129 | bta05203 | Viral carcinogenesis | 10 |
| 130 | bta05168 | Herpes simplex virus 1 infection | 14 |
| 131 | bta00220 | Arginine biosynthesis | 3 |
| 132 | bta04014 | Ras signaling pathway | 10 |
| 133 | bta00360 | Phenylalanine metabolism | 3 |
| 134 | bta04640 | Hematopoietic cell lineage | 6 |
| 135 | bta04670 | Leukocyte transendothelial migration | 6 |
| 136 | bta04261 | Adrenergic signaling in cardiomyocytes | 7 |
| 137 | bta04012 | ErbB signaling pathway | 5 |
| 138 | bta04935 | Growth hormone synthesis, secretion and action | 6 |
| 139 | bta04150 | mTOR signaling pathway | 7 |
| 140 | bta04611 | Platelet activation | 6 |
| 141 | bta05014 | Amyotrophic lateral sclerosis | 12 |
| 142 | bta04666 | Fc gamma R-mediated phagocytosis | 5 |
| 143 | bta05231 | Choline metabolism in cancer | 5 |
| 144 | bta04970 | Salivary secretion | 5 |
| 145 | bta04720 | Long-term potentiation | 4 |
| 146 | bta04928 | Parathyroid hormone synthesis, secretion and action | 5 |
| 147 | bta04140 | Autophagy - animal | 6 |
| 148 | bta05034 | Alcoholism | 8 |
| 149 | bta04622 | RIG-I-like receptor signaling pathway | 5 |
| 150 | bta05320 | Autoimmune thyroid disease | 4 |
| 151 | bta00061 | Fatty acid biosynthesis | 2 |
| 152 | bta04114 | Oocyte meiosis | 5 |
| 153 | bta00380 | Tryptophan metabolism | 3 |
| 154 | bta00590 | Arachidonic acid metabolism | 4 |
| 155 | bta04146 | Peroxisome | 4 |
| 156 | bta04310 | Wnt signaling pathway | 6 |
| 157 | bta04015 | Rap1 signaling pathway | 7 |
| 158 | bta04512 | ECM-receptor interaction | 4 |
| 159 | bta00340 | Histidine metabolism | 2 |
| 160 | bta04612 | Antigen processing and presentation | 4 |
| 161 | bta05032 | Morphine addiction | 4 |
| 162 | bta04360 | Axon guidance | 6 |
| 163 | bta04912 | GnRH signaling pathway | 4 |
| 164 | bta04810 | Regulation of actin cytoskeleton | 7 |
| 165 | bta04350 | TGF-beta signaling pathway | 4 |
| 166 | bta01212 | Fatty acid metabolism | 3 |

**Supplementary Table 9.** The Ramachandran plot values of the IL6 and IL10 model obtained using PROCHECK

| **Ramachandran plot value** | **Percentage of residues**  **Degree** | |
| --- | --- | --- |
|  | **IL6** | **IL10** |
| Most favoured regions | 75.8% | 92.3% |
| Additional allowed regions | 21.6% | 4.9% |
| Generously allowed regions | 2.0% | 2.8% |
| Disallowed regions | 0.7% | 0.0% |

**Supplementary Table 10.** The results of average daily gain, lung index and spleen index

| Index namen | Con | Mod | DSYXD | Dox |
| --- | --- | --- | --- | --- |
| Average daily gain (kg) | 1.89±0.35 | 0.21±0.27 | 0.57±0.11 | 0.45±0.05 |
| Lung index (%) | 1.1254±0.113 | 2.1641±0.104 | 1.5111±0.077 | 1.6301±0.138 |
| Spleen index (%) | 0.2196±0.020 | 0.2749±0.009 | 0.2387±0.008 | 0.1955±0.011 |

Notes: All values are listed with mean ± SD.

## Supplementary Figures


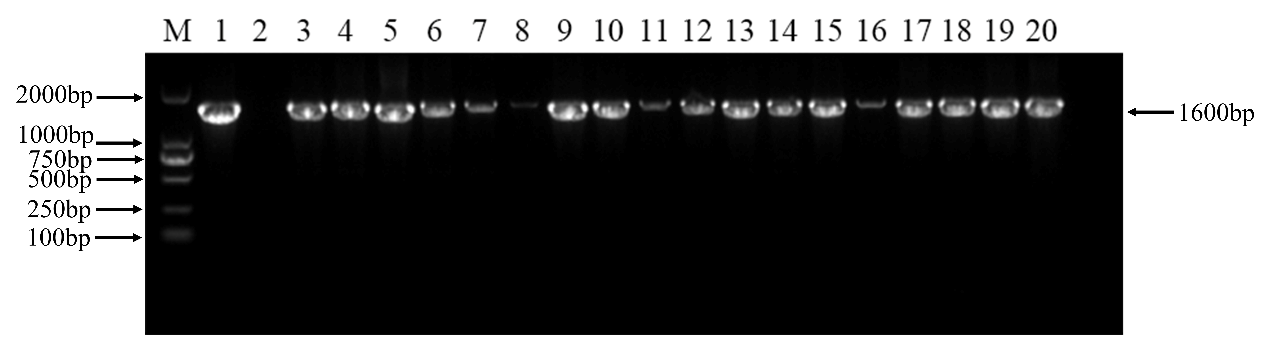


**Supplementary Figure 1.** UvrC primer PCR amplification results (M. DNA Marker DL 2000; 1. Positive control; 2. Negative control; 3~20. The nasal swab DNA-amplified products of pathology model calves)


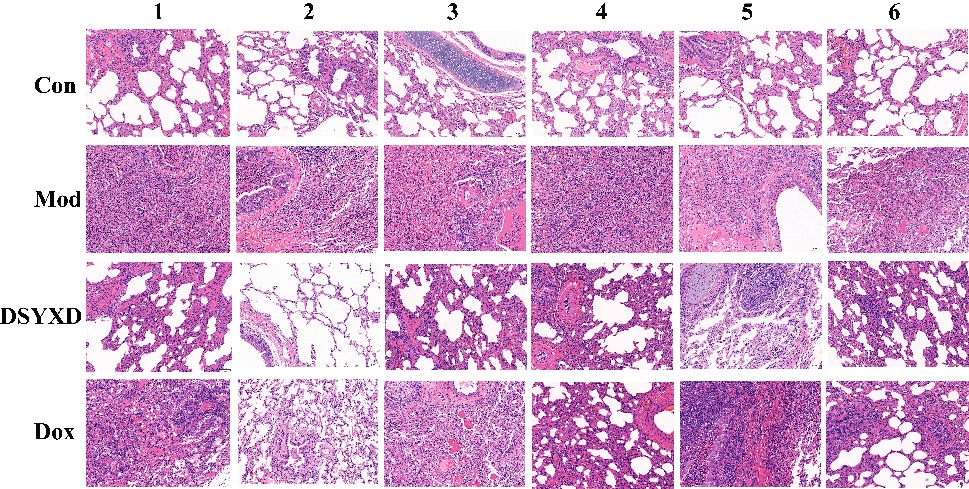


**Supplementary Figure 2.** HE staining of lung tissues (×200) [Control group (Con); model group (Mod); Dang-Shen-Yu-Xing decoction group (DSYXD); doxycycline group (Dox)]
